# Mesenchymal stem cell therapy for neurological disorders: The light or the dark side of the force?

**DOI:** 10.3389/fbioe.2023.1139359

**Published:** 2023-02-28

**Authors:** Jasmina Isaković, Klara Šerer, Barbara Barišić, Dinko Mitrečić

**Affiliations:** ^1^ Omnion Research International, Zagreb, Croatia; ^2^ Department of Histology and Embryology, University of Zagreb School of Medicine, Zagreb, Croatia; ^3^ University of Zagreb School of Medicine, Zagreb, Croatia; ^4^ University of Zagreb School of Dental Medicine, Zagreb, Croatia; ^5^ Laboratory for Stem Cells, Croatian Institute for Brain Research, University of Zagreb School of Medicine, Zagreb, Croatia

**Keywords:** mesenchymal stem cells, stem cell therapy, neurological disorders, ischemic stroke, multiple sclerosis, Alzheimer’s disease, Parkinson’s disease

## Abstract

Neurological disorders are recognized as major causes of death and disability worldwide. Because of this, they represent one of the largest public health challenges. With awareness of the massive burden associated with these disorders, came the recognition that treatment options were disproportionately scarce and, oftentimes, ineffective. To address these problems, modern research is increasingly looking into novel, more effective methods to treat neurological patients; one of which is cell-based therapies. In this review, we present a critical analysis of the features, challenges, and prospects of one of the stem cell types that can be employed to treat numerous neurological disorders—mesenchymal stem cells (MSCs). Despite the fact that several studies have already established the safety of MSC-based treatment approaches, there are still some reservations within the field regarding their immunocompatibility, heterogeneity, stemness stability, and a range of adverse effects—one of which is their tumor-promoting ability. We additionally examine MSCs’ mechanisms of action with respect to *in vitro* and *in vivo* research as well as detail the findings of past and ongoing clinical trials for Parkinson’s and Alzheimer’s disease, ischemic stroke, glioblastoma multiforme, and multiple sclerosis. Finally, this review discusses prospects for MSC-based therapeutics in the form of biomaterials, as well as the use of electromagnetic fields to enhance MSCs’ proliferation and differentiation into neuronal cells.

## 1 Introduction

### 1.1 Neurological diseases and disorders

Neurological disorders are disorders which affect the central and/or peripheral nervous system. Based on the pathophysiological mechanisms underlying their development, they can be classified into three major categories: 1) diseases where the symptoms can primarily be attributed to the loss of specific neurons or neuroglia, 2) diseases where cells are lost in non-specific ways as a consequence of acute damage, i.e., following the loss of circulation (stroke) or mechanical damage, and 3) diseases characterized by impaired function of neuronal cells, including the neuromuscular junction (NMJ).

The first category includes diseases like Parkinson’s and Alzheimer’s disease. While Parkinson’s is distinguished by the loss of a very specific cell subpopulation in a limited region of the brain, Alzheimer’s disease includes the loss of neurons in much wider anatomic regions, and at a rate which does not correlate to symptoms in a linear way. The chain of pathophysiological events that leads to cell loss in these cases is very complex and includes many types of molecules, particularly those that build up the cell projections, like tau (Vacchi et al., 2020). In addition, the pathology of many neurological disorders often includes some form of a specific immune or autoimmune reaction (e.g., in multiple sclerosis) prior to, or accompanying, the loss of neurons and neuroglia (Ibañez-Vega et al., 2021).

The second category of neurological disorders encompasses those accompanied by inflammation that develops following cell damage. These include stroke, traumatic brain injury (TBI) and traumatic spinal cord injury (SCI), among others. It is important to note that inflammation can, in these cases, bring both positive and negative effects (Peng et al., 2022). Finally, the third category includes diseases that are characterized by an impaired function of neuronal cells, including various epilepsies and disorders impacting the neuromuscular junction (e.g., myasthenia gravis) (San-Juan and Rodríguez-Méndez, 2022).

From a therapeutic point of view, our treatment approaches have always been only as successful as our characterization of the disease (e.g., myasthenia gravis or Parkinson’s disease). As a result, very specific drugs that boast higher efficiency rates have been developed for these. For example, pyridostigmine inhibits an enzyme that breaks down acetylcholine which, in turn, improves the propagation of signal to muscles in patients with myasthenia gravis (Lorenzoni et al., 2020). Similar successes have also been recorded with levodopa in patients with Parkinson’s disease, wherein the treatment directly increases the amount of the corresponding neurotransmitter whose quantities are often insufficient due to loss of neurons (Hauser, 2009). It is also worth mentioning that recent years have seen a rise in registration of completely new categories of drugs that improve the condition of diseases which have, thus far, been untreated (Zolgensma - gene therapy, and Spinraza—antisense oligonucleotide; both for treatment of spinal muscular atrophy) (Day et al., 2022). Despite these successes, we remain in a desperate need of drugs or other treatment modalities that would help address some of the more common neurological disorders, including stroke and Alzheimer’s disease. This is the case since their anatomical and pathophysiological complexity still represents obstacles which slow down the successful design of new drugs.

### 1.2 Mesenchymal stem cells (MSCs)

#### 1.2.1 Developmental origins

Mesenchymal stem cells (MSCs), or mesenchymal stromal cells, are a non-hematopoietic group of cell precursors that originate from mesoderm and ectoderm, the middle and outer embryonic germ layers, respectively. On top of being present in the developing embryo, where they migrate throughout the body during the process of maturation, MSCs can also be found, be it in small numbers, in some adult tissues. MSCs are viewed as prospective sources of allogenic cell therapy due to their multipotency, or the capacity to self-renew by dividing and differentiating into a wide variety of specialized cell types (Liu et al., 2022a).

The origin of the term “mesenchymal” refers to the mesenchymal tissue or embryonic connective tissue. These cells give rise to bones, cartilage, tendons, ligaments, muscles, and bone marrow, as well as express a variety of surface markers (vimentin, laminin B1, fibronectin, and osteopontin) (Caplan, 1991). Hence, MSCs’ ability to differentiate into a new cell lineage, or their “plasticity,” is one of their most intriguing characteristics (Dennis and Charbord, 2002). Interestingly, some studies also indicate that MSCs are genetically linked to a subset of ectoderm-derived cells called Sox-1 cells (Takashima et al., 2007). As such, current research suggests multiple developmental origins for the MSCs population. One potential pathway includes neural crest cells from the ectoderm. These cells express nestin and remain passive until the adult stage. The other pathway arrives from mesoderm, with a clear lack of nestin expression, and participates in creating the embryo skeleton. As a result, both the function and role of MSCs in adults are determined by their initial source (Isern et al., 2014).

The MSCs categorization standards for nomenclature, degree of stemness, and cell properties were released by The International Society for Cellular Therapy (ISCT) in 2006 (Andrzejewska et al., 2019). The name “multipotent mesenchymal stromal cells” was suggested as the most appropriate for the fibroblast-like plastic-adherent cells. Therein, MSCs are described as a population of cells that: 1) boast CD73, CD90 and CD105 surface antigen expression (coupled with a lack of CD45, CD34, CD14, CD11b, CD79a and CD19 expression), 2) adhere to plastic during cultivation, and 3) can differentiate into osteoblasts, adipocytes, and chondroblasts (Horwitz et al., 2005; Dominici et al., 2006; Andrzejewska et al., 2019).

Despite numerous decades of research into the MSC niche, our understanding of it remains quite limited. The term “niche” denotes a place in the body wherein stem cells are located, together with an environment around them that keeps these cells in an undifferentiated state. This definition is based on the hypothesis that the stem cells are undergoing constant interaction with other cells surrounding it that determine their behavior (Schofield, 1978). Analyzing all sites from which MSCs may be extracted, researchers proposed a notion that the MSC niche is found in blood vessels, which are present in all tissues appropriate for stem cell isolation (Putnam, 2014; Andrzejewska et al., 2019). This is supported by other studies revealing that some cells from the perivascular space (PVS) of blood vessels carry the markers CD146+, NG2+, PDGF-Rb+ and ALP+. After *in vitro* cultivation, these cells satisfy the requirements to be classified as MSCs (Crisan et al., 2008; Andrzejewska et al., 2019).

#### 1.2.2 Isolation and cultivation

**FIGURE 1:**
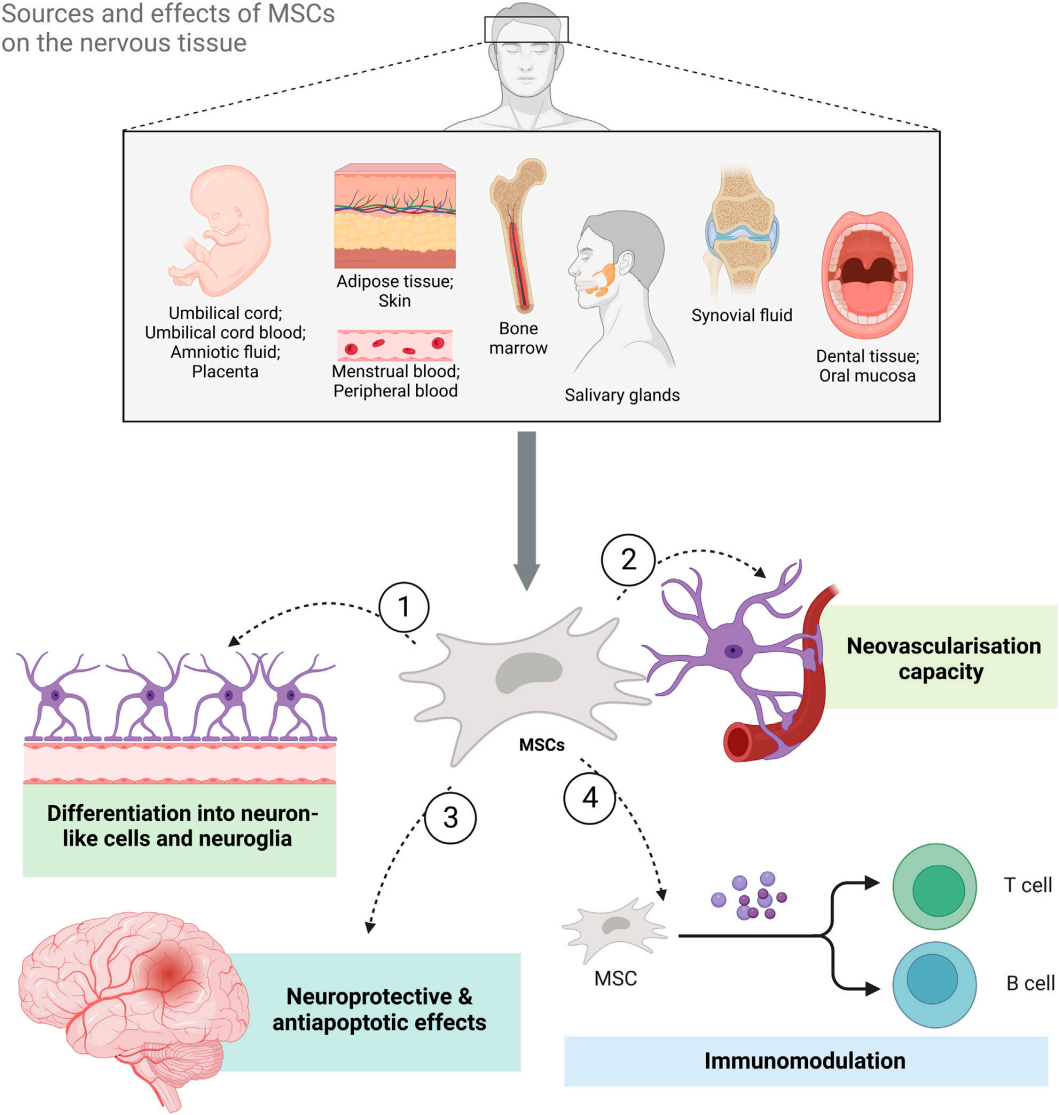
The sources of mesenchymal stem cells (MSCs) and the benefits of MSC-based therapy for neurological diseases and disorders. *MSCs can be isolated from both adult and perinatal tissue, umbilical cord, umbilical cord blood, amniotic fluid, placenta, adipose tissue, skin, bone marrow, salivary glands, synovial fluid, oral mucosa, and dental tissue as well as menstrual and peripheral blood. Their effects on neural tissue range from differentiation into neuron-like cells and neuroglia to immunomodulation and neovascularization. Additionally, they have also been shown to exert neuroprotective and antiapoptotic effects (Created with* *BioRender.com* *)*.

One of the most common hematopoietic stem cell sources in the human body, containing ample amounts of MSCs, is bone marrow (BM). Still, since the procedure used for collecting these cells is invasive, and brings discomfort to the patient, it is rarely used (Mushahary et al., 2018). Some other sources include: 1) umbilical cord (UC) (Salehinejad et al., 2012), 2) umbilical cord blood (UCB) (Lotfinejad et al., 2021), 3) adipose tissue (AT) (Pendleton et al., 2013), 4) amniotic fluid (AF) (’t Anker et al., 2003), 5) dental tissue (DT) (Huang et al., 2009), 6) skin (Riekstina et al., 2008), 7) placenta (P) (Miao et al., 2006; Raynaud et al., 2012), 8) salivary gland (Rotter et al., 2008), 9) synovial fluid (Hatakeyama et al., 2017), 10) oral mucosa (OM) (Alajbeg et al., 2018), menstrual blood (MB) (Nikoo et al., 2014; Zhao et al., 2018) and peripheral blood (PB) (Fu et al., 2012; Jain et al., 2020) (Figure 1). Due to the fact that they house large numbers of these cells, and present with a rather non-invasive collection method, UC and AT are highlighted as extremely significant sources of hMSCs (Mushahary et al., 2018).

Adipose-derived MSCs (AT-MSCs) can be easily isolated from adipose tissue, which is a regular surgical byproduct (Zuk et al., 2001), containing up to „3% stem and progenitor cells in its stroma-vascular fraction” (Mushahary et al., 2018). Since the frequency and yield of MSCs in AT is 2,500 times higher than that in BM (5 × 10^4^ to 2 × 10^5^ from 1 g vs 6–60 ×10^3^ from 1 ml, respectively) (Fraser et al., 2008), adipose tissue has been identified as a distinctly rich source of MSCs (Baer and Geiger, 2012). Nevertheless, MSCs are still more frequently isolated from the umbilical cord tissue (Wharton’s Jelly) and umbilical cord blood (Baer and Geiger, 2012), wherein the UC tissue represents the more abundant source of MSCs of the two (la Rocca et al., 2009). In addition, UC-MSCs are also significantly more multipotent than BM-MSCs or AT-MSCs (Kern et al., 2006).

On top of isolation of MSCs from AT and UC, one of the least invasive approaches towards autologous cells transplantation pertains to isolation of multipotent stem cells from the peripheral blood. While some studies have demonstrated that MSCs get mobilized into the peripheral blood in response to tissue damage in polytraumatized patients (Wiegner et al., 2018), others also detected their presence in patients with acute ST-elevation myocardial infarction (STEMI) (Pieper et al., 2017). Similarly, a 2020. study by Jain et al. has demonstrated that 42% of patients with multiple myeloma (MM) and lymphoma, together with healthy controls, boasted detectable concentrations of PB-MSCs (Jain et al., 2020). Since the authors reported no significant difference between the mean circulating MSCs and PB-MSCs on days 5 (p = 0.737) or 6 (p = 0.237), this suggests that peripheral blood appears to be a viable source for MSCs (Jain et al., 2020). Since PB-MSCs exhibit similar trilineage differentiation potential to that of BM-MSCs, as well as express lineage-specific genes, Fu et al. postulated that isolation of MSCs from the peripheral blood represents a feasible alternative to that from the bone marrow (Fu et al., 2012). Nevertheless, as these PB-MSCs are present in small numbers, their clinical use appears to only be feasible following *ex vivo* expansion (Jain et al., 2020).

MSCs are often extracted and cultured as a plastic-adherent cell population following a simple three-step procedure: 1) mincing of the tissue, 2) enzymatic digestion (optional), and 3) seeding and growth of cells on a plastic surface (Mushahary et al., 2018). Instead of the enzymatic procedure, researchers can also choose to follow an explant protocol. While the former involves enzymatic digestion and centrifugation of the tissue to achieve a single-cell suspension, the latter employs no enzyme but rather contains original tissue excised into smaller pieces (Hendijani, 2017). Although several groups utilize the enzymatic method for isolation of AT-MSCs, the use of the explant protocol provides less heterogeneous cell populations boasting higher viability and proliferation rates (Salehinejad et al., 2012). Since explant culture contains intact tissue pieces and undissociated extracellular matrix, this means that cells largely remain protected from proteolytic dissociation and mechanical stress (Hynes, 2009a; Jing et al., 2011a; Hendijani, 2017). On top of the beneficial environment explant culture creates for growing MSCs (Mushahary et al., 2018), it also facilitates: 1) enrichment of the cell medium through release of cytokines and growth factors (Hynes, 2009b), 2) higher cell yield (Jing et al., 2011b), 3) faster proliferation (Jing et al., 2011b; Shah et al., 2013), and 4) concurrent expression of surface markers like CD73, CD90, and CD105 (Priya et al., 2014).

## 2 Therapeutic potential and properties of MSCs

### 2.1 Differentiation into neuron-like cells and neuroglia

Not only do MSCs have the potential to differentiate into cells of the connective and muscle tissue, but also into non-mesodermal cells, particularly cells of neural lineage such as neurons and glia (George et al., 2019). Some studies also demonstrate that, when cultured in neural stem cell (NSC) culture conditions, MSCs can also form clusters called neurospheres, or even full functional units called organoids (Hernández et al., 2020). However, a challenge in the form of establishing communication between these cells and their subsequent production of neurotransmitters remains.

Due to their neuroprotective potential, and the important part they play during neurogenesis, MSCs can be utilized to treat many of neurological disorders. This especially holds true for MSCs that have been isolated from adipose tissue (AT-MSCs) (Bai et al., 2020) since they may develop into neuron-like cells and express distinct progenitor/mature neural markers (Urrutia et al., 2019). Therefore, the diseases for which MSCs present themselves as a potential treatment approach include various neurological injuries and diseases with an inflammatory etiology (Qu and Zhang, 2017).

Several studies have shown a significant benefit of MSCs in animal models of nervous system diseases as well as patients with neurological damage (Identifiers: NCT01771640, NCT01777646, NCT01895439, NCT01325103, NCT01325103, NCT02165904, and NCT02290886). One study pointed out the influence of bFGF and EGF in the differentiation of neural progenitor cells (NPCs) from MSCs (Khan et al., 2020). NPCs have the potential for terminal differentiation into neurons, boasting beta-III-tubulin expression, as well as the ability to initiate and conduct action potential. During the process of NPC differentiation, 1771 of the 3,252 genes shared by MSCs and NPCs were elevated, whereas 1,481 were downregulated (Khan et al., 2020). The most involved transcription factors noted in this study were Foxs1 and HEYL. This observation is consistent with the notion of “gap-junction-dependent cell to cell communication” which permits MSCs to differentiate into immature, neuron-like cells (Dilger et al., 2020). More details were clarified in study by Mareschi et al. who demonstrated that MSCs grown in a favorable medium began to express two K^+^ channels with two delayed rectifier K^+^ currents that are necessary for neuronal survival and basal cell activity (Mareschi et al., 2006). Additionally, Alizadeh et al. have shown that olfactory mucosa and Wharton’s Jelly can be used as sources of MSCs, specifically for differentiation into dopaminergic neurons and, therefore, as a viable treatment for Parkinson’s disease (Alizadeh et al., 2019). Similarly, other studies have demonstrated that both hDT-MSCs and hOM-MSCs can differentiate into dopaminergic-like neurons and, thereby, improve behavioral deficits and motor function in hemi-Parkinsonian rats (Ganz et al., 2014; Zhang et al., 2018a).

On the other hand, some researchers are also advocating for the use of MSCs isolated from the peripheral blood. Boasting a high neurogenic potential, coupled with a non-invasive isolation procedure, PB-MSCs have been proposed as an alternative MSC-based approach towards regeneration of neural tissue in both animal models (Fu et al., 2012) and humans (Ratajczak et al., 2011; Scapin et al., 2016; Barbon et al., 2021). These cells exhibit a high potential for neuronal differentiation (Ratajczak et al., 2011) as well as express several antigens that are characteristic for MSCs (Cesselli et al., 2009). Following neurogenic induction, they also boast dendrite-like morphology and exhibit distinct neuronal marker expression at levels of both mRNA and proteins (Barbon et al., 2021).

### 2.2 Immunomodulation

MSCs have wide range of immunomodulatory properties *via* the direct contact with immune cells and local environmental factors through their paracrine activity (Müller et al., 2021). Studies have shown that this effect occurs mainly due to MSC-secreted cytokines, chemokines, extracellular vesicles, and inflammatory stimuli. Herein, they seem to exert the main influence directly onto regulatory T-cell (Tregs) and monocytes (Song et al., 2020). Interestingly, even the apoptotic, metabolically inactivated, and fragmented MSCs have an immunomodulatory potential (Song et al., 2020).

**FIGURE 2:**
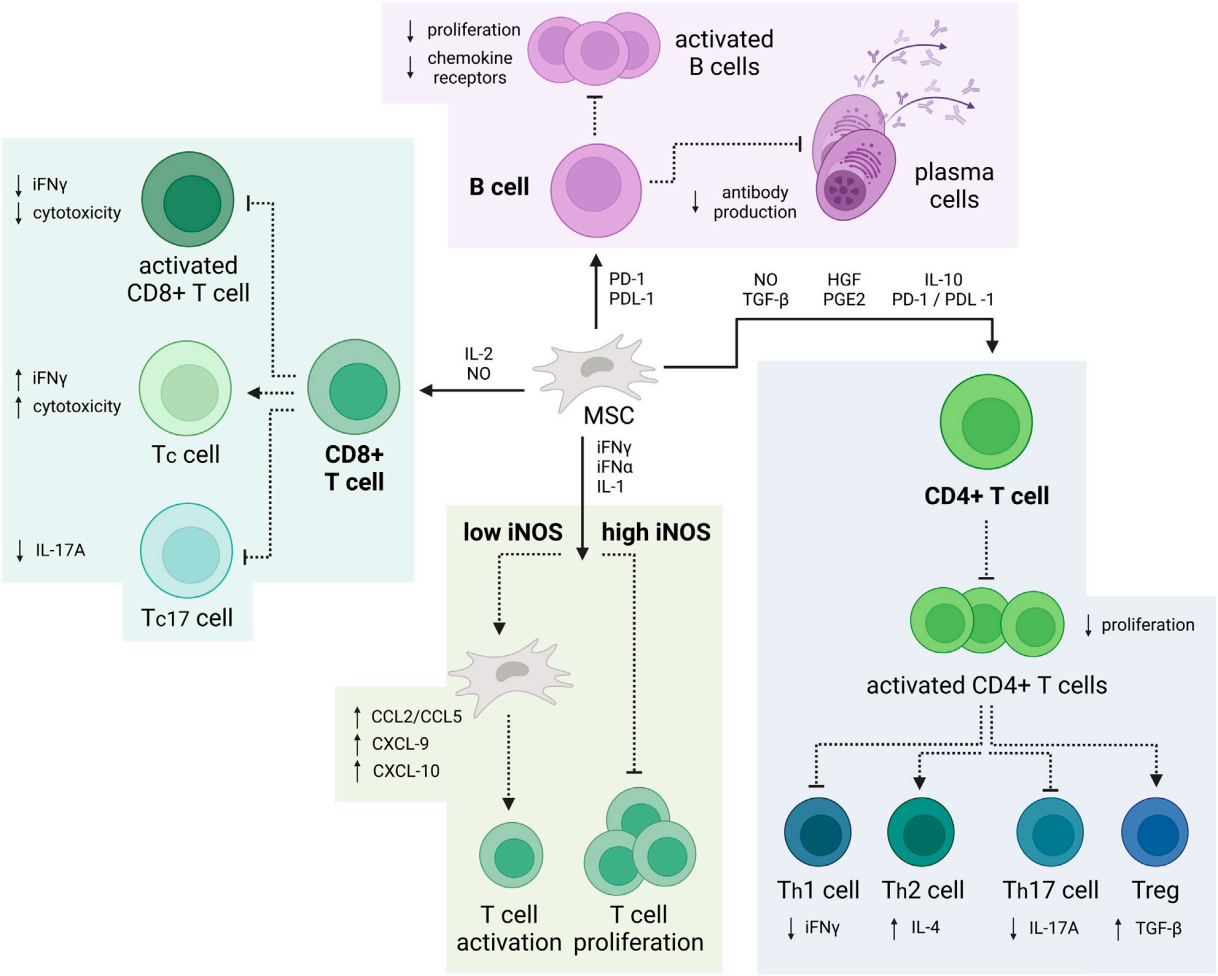
Immunomodulatory properties of MSCs. *MSCs can suppress the production of CD4* ^*+*^ *Th1 and Th17 while promoting proliferation of Tregs. Additionally, MSCs can impair the cytotoxic activity of proinflammatory CD8* ^*+*^ *T-cell and B-cell. Depending on the iNOS concentration, MSCs can also enhance or inhibit T-cell activation and proliferation (Created with* *BioRender.com* *).*

MSC-mediated immunomodulation is governed by various distinct cellular and molecular mechanisms (Weiss and Dahlke, 2019) (Figure 2). Through regulation of time-dependent release of NO, TGF-β, HGF, PGE2, IL-10 and PD-1/PDL-1, MSCs can inhibit CD4^+^ Th1 and Th17 cell activity as well as promote proliferation of Tregs, thereby enhancing their inhibitory capabilities (Wang et al., 2008; Ge et al., 2010; Luz-Crawford et al., 2019; Chen et al., 2020). The attenuation of Th1 and Th17 cell activity results in a decreased iFNγ and IL-17A release, slowing the immune response. Since IL-17 not only plays a role in clearance of extracellular but also intracellular pathogens, it is implied in pathogenesis of various autoimmune disorders (Kuwabara et al., 2017). Contrastingly, overactivation of Th2 and Tregs causes subsequent release of IL-4 and TGF-β, combination of which can induce microglial polarization towards their alternative (M2) phenotype (Zhou et al., 2012). Even though the specific function of this phenotype is still under debate, most research suggests that M2 microglia play a role in downregulation of the inflammatory response and assist in tissue repair (Cherry et al., 2014).

Similarly, MSCs can also suppress the cytotoxic activity of proinflammatory CD8^+^ T-cell, also known as cytotoxic T-cell (Tc17) through release of IL-2 and NO. This leads to a decrease in IL-17A and attenuates the immune response. Furthermore, depending on the iNOS concentration—be it high or low, MSCs can also inhibit T-cell proliferation or increase T-cell activation, respectively. Additionally, through release of PD-1 and PDL-1, MSCs limit B-cell differentiation, proliferation, and antibody release, all while promoting the development of IL-10-producing regulatory B-cell (Bregs) (Wehner et al., 2009; Deng et al., 2016; de Witte et al., 2018; Müller et al., 2021).

Like other properties of MSCs, their immunomodulatory capability depends on their origin. For example, even though some studies suggested that AT-MSC application in immunomodulatory therapy would yield better results than BM-MSCs, these findings are conflicting with other research within the field and, as such, remain inconclusive (El-Badawy et al., 2016; Li et al., 2019a; Adolfsson et al., 2020). On the other hand, since UC-MSCs have been shown to possess a low risk for invoking an allogeneic immune response they have entered the spotlight in recent years (Kim et al., 2018a; Song et al., 2020; Chen et al., 2021a).

### 2.3 Neuroprotective and anti-apoptotic effects

Human umbilical cord mesenchymal stem cells (hUC-MSCs) can generate a range of cytokines and neurotrophic factors, thereby promoting neuroregeneration (Xu et al., 2019a). In their study, Xu et al. found that transplantation of hUC-MSCs into neonatal rats resulted in decreased tissue damage and infarct volume due to cell migration into the periventricular tissue space (Xu et al., 2019a). Subsequently, this treatment resulted in improved motor function in neonatal rats. Moreover, hUC-MSCs have been shown to drastically lower apoptosis and the expression of Beclin-2 and caspase-3 (Xu et al., 2019a), the most critical apoptosis executors of the caspase cascade (Chang et al., 2005).

Additionally, Zhang et al. demonstrated that hMSCs’ anti-apoptotic effects are mediated by the apoptosis-regulating pathways involving Bcl-2 which, in low doses, provides neuroprotection against ischemic stroke (Zhang et al., 2019). On the other hand, application of hMSCs in medium to high doses has been shown to diminish their neuroprotective abilities. During this experiment, cocultures of primary rat neurons and astrocytes were placed in oxygen-glucose distress (OGD), followed by a 2-h period of reperfusion (Zhang et al., 2019). The cells were then treated with hBM-MSCs and/or a Bcl-2 antibody. The results have shown that implanted cells exhibit neuroprotective effects against stroke *in vitro via* an anti-apoptotic mechanism, which was blocked by the Bcl-2 antibody. Interestingly, administration of the Bcl-2 antibody alone had no effect on the viability of rat cells. Supporting this finding is a study by Calio et al. which shows that anti-apoptotic effects of MSCs can be impeded through inhibition of the Bcl-2 gene (Calió et al., 2014).

Many other studies have also shown that MSC-based therapies appear to promote the restoration of function in cases of hypoxic–ischemic brain damage (HIBD) by exerting immunomodulatory effects (Gu et al., 2015). Research by Gu et al. focused on explaining the mechanisms underlying MSC-mediated immunomodulation through Toll-like receptor 2 (TLR2) and interleukin-10 (IL-10) (Gu et al., 2015). Since TLRs mediate the tissue response to pathogenic processes and injury, they get activated in neurons under ischemia (Kawai and Akira, 2006; Lehnardt et al., 2007). IL-10, on the other hand, limits the production of pro-inflammatory cytokines and chemokines, thereby suppressing the immune response (Moore et al., 2001). With that, the findings presented by Gu et al. point to TLR2’s involvement in the pathogenesis of HIBD, with application of MSCs decreasing apoptosis and improving cognitive functions in HIBD rats (Gu et al., 2015). This is accomplished through suppression of the TLR2/NFκB signaling pathway, with the help of a feedback mechanism that diminishes subsequent IL-10 release. Additionally, our group has also demonstrated that hOM-MSCs exert neuroprotective effects on cells cultured in anoxic conditions and mice with middle cerebral artery occlusion (MCAO) through time-limited secretion of miR-514A-3p (Stančin et al., 2022).

### 2.4 Neovascularisation capacity

Stroke is one of the leading causes of mortality worldwide (Mathers et al., 2009)—with ischemic stroke (IS) accounting for 87% of those cases (Towfighi and Saver, 2011). Nevertheless, therapeutic options remain limited and include: 1) intravenous injection of tissue plasminogen activator (t-PA) (Del Zoppo et al., 2009), and 2) mechanical thrombectomy (MT) (Nogueira et al., 2018). Interestingly, the vast majority of research throughout the years has demonstrated that MSCs may minimize the extent of cerebral infarction following ischemia and, subsequently, induce some restoration of function (Onda et al., 2008; Nakajima et al., 2017; Huang et al., 2018; Zhou et al., 2019; Huang et al., 2021). As such, the specific therapeutic mechanism of MSCs in ischemic stroke may be related to their ability to promote neurogenesis, angiogenesis, and exert anti-inflammatory effects (Li et al., 2021a; Chen and Zhou, 2022; Zhou et al., 2022).

Ischemic stroke leads to destruction of capillaries and causes an increase in blood-brain barrier (BBB) permeability. This further amplifies the inflammatory response, necrosis of neurons, and brain edema (Guo et al., 2021). Following stroke, neovascularization aids in reestablishing the blood and oxygen supply, thereby facilitating nerve regeneration (Guo et al., 2021). For example, Krupinski et al. discovered that microvessel density in the ischemic area is positively corelated with the patients’ survival (Krupinski et al., 1994). Similar results have been obtained in a rat model by Kan et al. wherein they observed that microvascular remodeling corresponded to tissue edema status (Kang et al., 2020). Combined, these studies demonstrate that poststroke revascularization is critical for establishing a positive clinical outcome (Talwar and Srivastava, 2014; Rust, 2020; Ma et al., 2021a).

It is believed that poststroke transplantation of MSCs enhances angiogenesis by producing or boosting endogenous nutrients (Krupinski et al., 1994)—including vascular endothelial growth factor (VEGF) (Huang et al., 2014), angiopoietin-1 (Ang-1) (Onda et al., 2008), placental growth factor (PlGF) (Liu et al., 2006), and basic fibroblast growth factor (bFGF) (Ghazavi et al., 2017). In turn, the presence of these nutrients can aid the development of the immature vascular trunk (Carmeliet and Collen, 1997) or assist in vessel maturation (Carmeliet and Collen, 1997; Suri et al., 1998), thereby reducing the infarct size. Moreover, MSCs have been shown to aid in angiogenesis by engaging the Notch signaling cascade in the endothelial cells (Guo et al., 2012). This could be associated with endothelial cell production of VEGF-A (Zhu et al., 2015). Admittedly, the application of dual antiplatelet therapy (DAPT), which inhibits the Notch pathway, led to a decrease in VEGF-A and the inhibition of angiogenesis following transplantation of MSCs (Zhu et al., 2015). Furthermore, Hong et al. found that coculturing of MSC culture supernatant with human aortic endothelial cells results in inhibition of the hypoxia-induced apoptosis and promotion of angiogenesis through activation of the PI3K Akt signaling pathway (Shih-Chieh Hung et al., 2007).

On the other hand, the functional stability of brain microvascular endothelial cells (BMVECs) is also important for ensuring the BBB consistency (Yang and Rosenberg, 2011). A study by Chung et al. has shown that intravenous administration of hAT-MSCs reduces BBB leakage in stroke rats by preventing disruptions in its structure and diminishing the damage of the endothelial vasculature (Chung et al., 2015). On top of exerting direct action, transplantation of MSCs can also mediate intracellular communication through release of extracellular vesicles. These EVs contain annexin A1 (ANXA 1), an anti-inflammatory agent that is expressed in BMVECs and microglia (McArthur et al., 2016; Chen et al., 2017; He and Ye, 2017; Maia et al., 2017). Not only does ANXA1 protect neurons from injury (Luo et al., 2014), but it also plays a role in BBB integrity, as demonstrated by Gussenhoven et al. (Gussenhoven et al., 2019). Finally, MSCs have also been implicated in suppression of VEGF-induced BBB leakage through uptake of glucose from endothelial cells, all while inhibiting VEGF absorption (Do et al., 2021; Kikuchi-Taura et al., 2021).

### 2.5 MSCs as therapeutic carriers

Some research performed throughout the past decade has also investigated stem cells’ ability to act as therapeutic carriers, meant to facilitate targeted delivery of a variety of pharmacological agents. Due to their intrinsic property, wherein stem cells are attracted to tumor sites, this approach is promising in delivery of oncolytic viruses as potential anticancer therapeutics (Litvinova et al., 2022), or a variety of other application relating to cancer (Hmadcha et al., 2020a). Additionally, these MSCs could also be used for designing targeted treatments for glioblastoma, astrocytoma and oligodendrocytoma as well as Parkinson’s and Alzheimer’s disease.

One of the more novel approaches to cancer treatment includes loading of MSCs with oncolytic viruses (Hmadcha et al., 2020b). For example, the loading of MSCs with the oncolytic herpes simplex virus provided them with the ability to detect metastases, increasing the lifespan of mice with brain metastatic melanomas (Du et al., 2017). Additionally, other studies performed loading of MSCs with anticancer drugs, resulting in antitumor effects in glioma-bearing rats (Pacioni et al., 2015).

Since there exist many methods for encapsulating the biomolecules containing bioactive materials or anti-cancer drugs, recent studies have been centered around the discovery of strategies for optimization of the payload and delivery capacity of MSCs (Hmadcha et al., 2020b). While some utilized nanoparticles to increase the anti-tumor efficacy of MSCs (Layek et al., 2018; Wang et al., 2018; Moku et al., 2019; Cheng et al., 2021; Ebrahimian et al., 2022), others performed microcapsule loading of MSCs for the purpose of testing their ability to serve as a delivery vehicle across a variety of tissue-blood or tumor-blood barriers (Litvinova et al., 2022). For example, a study by Litvinova et al. has shown that loading of hMSCs with synthesized microcapsules causes no damage to the cell’s structural integrity (Litvinova et al., 2022). Furthermore, this method also yielded cells with preserved motility and the capacity to migrate through 8 μ m pores.

### 2.6 Application of extracellular vesicles derived from MSCs

Exosomes are membrane-bound, nano-sized extracellular vesicles (EVs) generated in many eukaryotic cells’ endosomal compartments. Since they carry nucleic acids, proteins, lipids, and a variety of metabolites, they are mediators of both near and long-distance intracellular communication. Exosomes aid this intracellular communication by transporting lipids, proteins, RNA, miRNA, and membrane receptors (Raposo and Stoorvogel, 2013). This mechanism represents a viable therapeutic strategy for a variety of neurological diseases and disorders (Gorabi et al., 2019a). With that, MSC-derived exosomes have been shown to play important roles in immunomodulation and tissue repair (Gorabi et al., 2019b). Specifically, WJ-MSC-derived exosomes may rescue the process of myelination, inhibit oligodendrocyte and neuron cell death, and reduce microglia-mediated neuroinflammation (Thomi et al., 2019a; Thomi et al., 2019b).

Even though the mechanism behind this occurrence is yet to be elucidated, there is a plethora of reports demonstrating that exosomes can cross the BBB (Banks et al., 2020). This addresses one of the most prominent challenges in treatment of neurological disorders—the inability to facilitate direct delivery of therapeutic agents through the BBB. While immune cells and viruses are often transported over the BBB *via* several neuroimmune mechanisms (Banks et al., 2001; Turowski et al., 2005; Wolburg et al., 2005), recent research indicates that some of these mechanisms are also involved in the transport of the exosomes across the BBB (Wurdinger et al., 2012), including endocytic (vesicular) processes (Chen et al., 2016).

On top of directly utilizing EVs derived from MSCs, there also exist attempts to manipulate the EVs *ex vivo* in order to facilitate a more optimal therapeutic approach. One of the most used methods for manipulation of EVs includes the modulation of their miRNA content (Yoo et al., 2018; Abreu et al., 2021; Zeng et al., 2022). This is performed by a procedure termed exo-miRNA loading, which is done in one of two ways: 1) producing a cell line over-expressing the desired miRNA or 2) loading EVs with miRNA using chemical or physical methods (Munir et al., 2020). While the first approach includes the secretion of miRNA-loaded EVs by an over-expressing cell line that is exhibiting high miRNA concentrations in its cytosol, the second approach takes advantage of the electrochemical properties of miRNAs and loads them into the exosomes *via* sonication or electroporation (Weaver, 1993). The latter process is associated with alterations in the structure’s innate thermal energy and transient elevation of the transmembrane potential. This leads to rapid, but localized, rearrangement of the molecular structure of the membrane and creation of hydrophilic pores (Weaver, 1993; Chen et al., 2006). These miRNA-enriched EVs can then be used to selectively target a variety of cell types.

Correspondingly, exosomes could also be adapted for therapeutic use through modification of their surface and membrane proteins (Munir et al., 2020). This facilitates enhanced cell-selectivity and decreased clearance rate within the target tissue. Modification of the exosome’s surface and membrane proteins can be performed either by direct methods or genetic alterations. While the direct method involves the mixing of the protein with the exosomes (non-covalent method) or attaching of the peptide with covalent linkage (covalent method), genetic alteration includes the modulation of the cells to produce a particular protein on the exosome surface (Munir et al., 2020). As opposed to electroporation, both direct methods and genetic alterations of the exosomes have a few prominent disadvantages, namely, chemical contamination and variable efficiency (Hu et al., 2020) or safety concerns owing to potential immune responses (Ohno et al., 2013), respectively.

Nevertheless, a great deal of advancement has been achieved throughout the past decade with respect to application of MSC-derived EVs and exosomal miRNAs in treatment of a variety of neurological disorders (Xin et al., 2012; Nakano and Fujimiya, 2021; Soni et al., 2022a; Yang et al., 2022). For example, following the purification of exosomes stemming from hBM-, hAD- and hWJ-MSCs, Soni et al. have shown that exosomes possess the ability to induce neurogenesis and angiogenesis (Soni et al., 2022b). Similar results have also been observed in animal models of Alzheimer’s disease (Reza-Zaldivar et al., 2019) and traumatic brain injury (Zhang et al., 2015), with enhanced animal recovery also visible in cases of spinal cord injury (Zhou et al., 2021).

## 3 The use of MSCs in neurology

### 3.1 Alzheimers’ disease

Alzheimer’s disease (AD) is a neurodegenerative disease that represents the most frequent cause of age-related dementia. With that, it exerts enormous financial, societal, and health burdens (Horgan et al., 2020). Although the pathophysiology of AD is still relatively unknown, some variants of the amyloid β (A β ) protein that accumulate in the extracellular matrix have been implied in its development. Since the current therapeutic options can only mildly reduce the symptoms of the disease, researchers are increasingly turning towards cell-based therapies (Table 1). As such, they are expecting that stem cells could decrease cell death, or even replace the damaged neurons with new ones. Another interesting property of stem cells, which is currently in the forefront of cell-based therapies for AD, is their ability to clear toxic aggregates. In this sense, stem cells could delay or even stop disease development.

**TABLE 1:** Summary of *in vitro* and *in vivo* studies investigating the beneficial effects of MSCs in Alzheimer’s disease.

Treatment	Disease model	Results	Mechanisms of action	Authors
NFT-SCs derived from AT-MSCs	SH-SY5Y cells treated with Aβ1-42	Decreased cell death	Paracrine and juxtacrine interactions	Jahed et al. (2021)
BM-MSCs	3xTg-AD mouse model	Reduced pathological Tau phosphorylation and decrease in inflammation	Modulation of cAMP-PKA activity	Neves et al. (2021)
			Increase in protein phosphatase 2 (PP2A) activity	
MB-MSCs	APP/PS1 Transgenic Mice	Reduced inflammation	Reduction of tau hyperphosphorylation through inactivation of GSK-3β	Zhao et al. (2018)
		Improved spatial learning and memory		
UC-MSCs	Tg2576 mice	Improved cognitive function	Increase in malondialdehyde (MDA), NO, SOD and nNOS	Cui et al. (2017)
		No changes in Aβ levels in the hippocampus		
		Reduced oxidative stress	Upregulation of silent information regulator 1 (Sirt1), BDNF and synaptophysin (SYN) levels	
AT-MSC-derived NFT-SCs	BALB/c mice	Improved learning and memory	Activation of the Wnt/β-catenin pathway through Trk *via* LRP6 phosphorylation downstream of the MAPK/ERK pathway	Bahlakeh et al. (2022)
		Increase in BrdU/Nestin+ and BrdU/NeuN + cells in SGZ of the hippocampus		
WJ-MSC-derived exosomes loaded with miR-29a	J20 mouse model of AD	Decrease in Aβ expression	Decrease in nuclear HDAC4	Chen et al. (2021b)
		Improved cognitive recovery		
BM-MSC-derived exosomes loaded with miR-29a	Rats injected with Aβ1-42 into the dorsal hippocampus (CA1)	Improvements in spatial learning and memory	Increase in endogenous miR-29b levels	Jahangard et al. (2020)
			Decrease in expression of NAV3 and BIM	
hBM-MSC-derived LMP1-exosomes	5XFAD mice	Improved cognitive performance	Not specified	Cone et al. (2021)
		Decreased Aβ plaque deposition in the hippocampus		
Hypoxia-preconditioned BM-MSC-derived exosomes	APP/PS1 Transgenic Mice	Rescued cognition and memory impairment	Downregulation of proinflammatory (TNF-α and IL-1β) cytokines	Cui et al. (2018)
		Reduced plaque deposition	Upregulation of anti-inflammatory cytokines (IL-4 and IL-10)	
		Restoration of synaptic dysfunction	Regulation of miR-21	

A recent publication by Neves et al. presented their data following MSC transplantation, wherein it was shown that MSCs reduce Tau phosphorylation and inflammation in an AD mouse model (Neves et al., 2021). Other publications have shown that MSCs can also reduce inflammation (Zhao et al., 2018) and improve cognitive function of AD affected mice (Cui et al., 2017). One of the mechanisms most probably involved in these improvements is the reduction of cell death (Zhang et al., 2020c).

Interestingly, some groups have also taken advantage of the proliferation and differentiation capacity of MSCs to alleviate the Alzheimer’s disease-like pathology in both *in vitro* (Jahed et al., 2021) and *in vivo* settings (Bahlakeh et al., 2022). A 2021. study by Jahed et al. utilized hAT-MSCs isolated from patients (20–40 years old) that underwent liposuction surgery and trans-differentiated them into neurotrophic factor-secreting stem cells (NTF-SCs) (Jahed et al., 2021). These cells, or their cell medium (CM), were then cocultured with the human neuroblastoma cell line (SH-SY5Y) treated with Aβ_1-42_, mimicking AD-like pathology *in vitro*. The results have shown that NFT-SCs decreased SH-SY5Y cell death as well as reduced Tau hyperphosphorylation and TNF-α levels, when compared to the control (*p* < 0.05). Additionally, these NFT-SCs have also been shown to release increased quantities of NGF and BDNF, when compared to MSCs (*p* < 0.05). Similarly, a study by Bahlakeh et al. transplanted hAT-MSC-derived NFT-SCs into BALB/c mice exhibiting an AD-like pathology (Bahlakeh et al., 2022). BALB/c mice treated with NFT-SCs and NFT-SCs CM exhibited improved learning and memory, as indicated by their performance in the Morris water maze (MWM) test. Namely, mice in both treatment groups exhibited significantly reduced swim and escape latency relative to the control (*p* < 0.05). Subsequently, the transplantation of NFT-SCs induced an increase in BrdU/Nestin^+^ and BrdU/NeuN^+^ cells in SGZ of the hippocampus. Generally, both studies appear to suggest that NFT-SCs enhance adult endogenous neurogenesis, alleviating some aspects of AD-like pathologies.

In addition to direct transplantation of MSCs, there also exist studies researching the application of MSCs’ byproducts, such as exosomes (Zeng et al., 2022). For example, a 2021. study by Chen et al. has shown that WJ-MSC-derived exosomes loaded with miR-29a, which mainly affects histone deacetylase 4 (HDAC4) (Müller et al., 2016), caused a significant decrease in nuclear HDAC4 (Chen et al., 2021b). This lowered the expression of A β and improved cognitive recovery in AD mice. Similar results with miR-29b-enriched EVs were also obtained by Jahangard et al. in rat AD models (Jahangard et al., 2020). On top of miR-29, studies have also shown beneficial effects of miR-21, miR-22, miR-1246, wherein these miRNAs slowed cognitive deterioration in AD mice through regulation of neuroinflammation and synaptic damage (Cui et al., 2018; Cone et al., 2021). With that, in order to optimize miR cargo delivery and, therewith, develop novel AD treatments, one must first understand the intricate connection between progression of AD and exo-miRs (Zeng et al., 2022).

Finally, many clinical trials pertaining to the application of MSCs in treatment of AD have taken place during the past decade. Most of these have been phase I trials that tested the safety of application of MSCs in patients affected by AD. Apart from finding that this approach is safe and well tolerated, some clinical trials have also reported several beneficial effects of transplanted MSCs. One of the trials, performed on 33 patients in the United States, included an intravascular administration of MSCs (Identifier: NCT05233774) (Brody et al., 2022). The preliminary results show significant increase in the hippocampal volume within the treatment group, coupled with improvements in neurocognitive scores as compared to the placebo.

### 3.2 Parkinson’s disease

Parkinson’s disease (PD) is a progressive condition marked by neurodegeneration of the substantia nigra pars compacta (SNpc) (Song and Kim, 2016). When it comes to its treatment, MSCs have demonstrated significant potential in targeting the disease’s pathogenesis through: 1) influencing the symptoms, 2) modifying the course of the disease, and 3) controlling disease manifestation (Table 2). A study by Boika et al. confirmed that the transplantation of MSCs into PD patients decreased the severity of motor and non-motor symptoms (Boika et al., 2020), suggesting that MSCs are interesting potential candidates for disease-modifying strategies in PD (Vilaça-Faria et al., 2019; Berlet et al., 2022; Heris et al., 2022).

**TABLE 2:** Summary of *in vitro* and *in vivo* studies investigating the beneficial effects of MSCs in Parkinson’s disease.

Treatment	Disease model	Results	Mechanisms of action	Authors
BM-MSCs transfected with Notch intracellular domain (NICD) and subsequent GDNF administration	Rat model of PD	Increase in proportion of TH-positive and dopamine-producing cells	Action of CNTF *via* the MAPK pathway	Dezawa et al. (2004)
		Functional improvements	Synergistic effect toward neuronal induction when combined with bFGF and FSK stimuli	
NSCs cultured in CM containing MSCs secretome	Dopa-deficit model rats	Behavioral improvements	Differentiation of CM-NSCs into dopaminergic neurons of the VTA and MFB	Yao et al. (2016)
A9 dopaminergic neuron-like cells induced from MSCs	Hemiparki-nsonian macaques	Modest and gradual improvements in motor behaviors	Not specified	Hayashi et al. (2013)
		Persistence of A9 dopaminergic neuron markers in the engrafted striatum at 9-month checkup		
hUC-MSCs	MPTP-induced mouse model of PD	Alleviated locomotor deficits	Changes in the brain–gut axis	Sun et al. (2022b)
		Rescued dopaminergic neurons through inhibition of neuroinflammation		
Neuronal-primed hBM-MSCs	Hemiparki-nsonian rats	Differentiation of hBM-MSCs into immature neuron-like cells	Not specified	Khoo et al. (2011)
		Only transient survival and engraftment of hBM-MSCs observed		
hBM-MSCs	6-OHDA rat model	Improvement in behavioral deficits	Functional cell replacement	Levy et al. (2008)
		Survival of grafted cells with dopaminergic traits for over 130 days following transplantation		
hBM-MSCs	6-OHDA rat model	Behavioral improvement 3 months following cell transplantation	Transdifferentiation into functional dopaminergic neurons	Shetty et al (2009)
NFT-SCs derived from BM-MSCs	6-OHDA rat model	Behavioral improvements visible in decrease in amphetamine-induced rotations	Immunomodulation and secretion of trophic factors	Sadan et al. (2009)
GDNF-transduced BM-MSCs	6-OHDA rat model	Behavioral improvements	Local trophic effect in the denervated striatum	Moloney et al. (2010)
			Induction of sprouting from the dopaminergic terminals towards the created neurotrophic milieu	
BM-MSCs	6-OHDA rat model	Behavioral improvements	Partial restoration of the dopaminergic markers and vesicular striatal pool of dopamine	Bouchez et al. (2009)

One technique pertaining to MSC transplantation in PD patients is described by Dezawa et al. (Dezawa et al., 2004). While autocell transplantation includes the use of BM-MSCs isolated from the patients themselves, allocell transplantation requires AT-, BM- or UC-MSCs obtained from healthy donors (Kitada and Dezawa, 2012). On the other hand, Yao et al. have utilized NSCs cultured in a conditioned medium (CoM) containing the MSC secretome for transplantation into dopa-deficit model rats (Yao et al., 2016). Their findings show that CoM-NSCs differentiate towards dopaminergic neurons of the ventral tegmental area (VTA) and the medial forebrain bundle (MFB) (Mendes Filho et al., 2018). When conditioned, these cells also exhibited higher survival and migration as well as induced significant behavioral improvements, when compared to untreated NSCs. Even though advances are being made daily, cell transplantation *via* intravenous administration still has a high risk of causing pulmonary thrombosis (Ramot et al., 2010).

On a molecular level, MSC-based treatment for PD has two distinct effects: 1) trophic action driven by cytokines and numerous neuroprotective, anti-apoptotic, and growth factors (Hofer and Tuan, 2016; Fu et al., 2017; Kim et al., 2018b) and 2) differentiation of MSCs into a variety of distinct cell types facilitating cell replenishment (Alizadeh et al., 2019; George et al., 2019; Hernández et al., 2020). Additionally, MSCs have also been reported to release anti-inflammatory cytokines and, thereby, aid in tissue healing (Sun et al., 2022a). Even though some studies demonstrated that MSCs have the potential to differentiate into DA neuronal precursors, it remains unknown whether these differentiated cells can be assimilated within the host environment and forge new synaptic connections with the host neurons (Hayashi et al., 2013; Zeng et al., 2015a). According to Fričova et al., MSCs are the more prominent forerunners for cell-based therapy due to their regenerative and immunomodulatory potential, minimal risk of tumor development, and sparse ethical concerns (Fričová et al., 2020).

Recent studies have also shown that MSCs-derived secretome has therapeutic effects in PD by secreting a variety of soluble factors and encapsulated extravesicles, all while avoiding the allogenic immune response (Ferreira et al., 2018; Phelps et al., 2018; Samanta et al., 2018). These exosomes can cross the BBB and exert neuroprotective effects (D’angelo et al., 2020). As such, MSCs therapy in combination with Levodopa might be the future standard of PD treatment (Heris et al., 2022).

Some application of MSCs for treatment of PD have also entered clinical trials to compare their efficacy (Identifiers: NCT03550183, NCT01446614, NCT02611167, NCT04506073, NCT03684122, NCT04146519, NCT04928287, NCT04876326 and NCT04995081) (Liu et al., 2022b). No results have been reported yet. Nonetheless, since the number of active clinical trials is modest, further investigations are required to assess the safety and efficacy of MSC-based therapies for PD.

### 3.3 Multiple sclerosis

Multiple sclerosis is a chronic inflammatory and neurodegenerative disease of the CNS characterized by lesions of the white matter, mostly manifesting through damage to the myelin sheath and axons. Following an attack by autoreactive T-cell and endogenous remyelination failure, MS leads to neurological dysfunction (Genc et al., 2019). Since the complex pathogenesis of MS is not yet completely understood, current immunosuppression-based therapies have a low efficiency. As such, alternate approaches to MS management and treatment are being researched throughout. One of the more prominent candidates for this new form of treatment are MSCs and associated exosomes (Table 3). Exosomes (and other EVs) derived from MSCs have been demonstrated to ameliorate motor function impairments in models of multiple sclerosis, decrease the proinflammatory response, and reduce demyelination (Zappia et al., 2005; Gerdoni et al., 2007; Kassis et al., 2008; Lu et al., 2009; Rafei et al., 2009).

**TABLE 3:** Summary of *in vitro* and *in vivo* studies investigating the beneficial effects of MSCs in multiple sclerosis.

Treatment	Disease model	Results	Mechanisms of action	Authors
hAT-MSC-derived EVs	SJL/J mice with TMEV-induced demyelinating disease	Diminished brain atrophy	Modulation of the microglial activation state	Laso-García et al. (2018)
		Increased cell proliferation	Reduction in plasma cytokine levels (Th1 and Th17)	
		Decreased inflammatory response		
		Improved motor deficits		
hP-MSC-derived EVs	EAE mouse model	Improved motor function	Differentiation of endogenous oligodendrocyte precursor cells into mature myelinating oligodendrocytes	Clark et al. (2019)
hBM-MSCs stimulated by IFN-γ	EAE mouse model	Improved motor function	Increase in the number of CD4^+^CD25+FOXP3+ Tregs	Riazifar et al. (2019)
		Decreased demyelination and inflammation	Reduction in the total number of macrophages/microglia and pro-inflammatory T-cell	
hBM-MSC-derived EVs	EAE rat model	Behavioral improvement	Regulation of microglial polarization from M1 to M2	Li et al. (2019b)
		Reduced infiltration of inflammatory cells		
		Decreased demyelination		
hDT-MSC-derived EVs	EAE mouse model	Attenuation of the inflammatory response	Inactivation of the NALP3 inflammasome	Soundara Rajan et al. (2017)
		Decreased infiltration of inflammatory cells	NF-kB reduction	
hDT-MSC-derived MVs	EAE mouse model	Alleviation of disease progression	Inhibition of activation and proliferation of lymphocytes	Mokarizadeh et al. (2012)
			Secretion of anti-inflammatory cytokines	
Recombinant interleukin 23 receptor (RIL-23R)-engineered MSCs	EAE mouse model	Increased myelination	Suppression of T lymphocyte proliferation	Rostami et al. (2022)
		Alleviation of disease progression	Reduction in penetration of inflammatory cells in the white matter	

Following intravenous administration of EVs derived from hAT-MSCs into SJL/J mice with Theiler’s murine encephalomyelitis virus (TMEV)-induced demyelinating disease, Laso-García et al. have demonstrated several beneficial effects to the host tissue. Namely, they observed diminished brain atrophy, increased cell proliferation, decreased inflammatory response, and improved motor deficits (Laso-García et al., 2018). Firstly, a clear reduction in neuroinflammation was observed, visible in reduced GFAP and Iba-1 staining, accompanied by an increase in myelin protein expression in the brain (Laso-García et al., 2018). Changes were also observed in the spinal cord, manifesting as distinct alterations in the morphology of the microglial cells, suggesting that EVs might be able to modulate the activation state of microglia. Immunomodulatory ability of EVs was visible in reduction in plasma cytokine levels, mainly that of Th1 and Th17.

On the other hand, Clark et al. have utilized the experimental autoimmune encephalomyelitis (EAE) murine model of MS to show that human placental MSC (hP-MSC)-derived EVs promote remyelination (Clark et al., 2019). They demonstrated that, as opposed to the control group, animals treated with hP-MSCs and hP-MSC-EVs displayed increased “differentiation of endogenous oligodendrocyte precursor cells into mature myelinating oligodendrocytes” (Mukai et al., 2021), resulting in an improved motor function in those groups. Furthermore, mice in these groups exhibited less DNA damage to oligodendrocytes.

Confirming similar results in EAE mice, Riazifar et al. have performed intravenous administration of hBM-MSCs stimulated by IFN-γ (Riazifar et al., 2019). Their research found that application of hBM-MSCs reduced the mean clinical score of EAE mice, diminished demyelination and inflammation, and increased the number of CD4^+^CD25+FOXP3+ Tregs. More recent studies on EAE mice, following intravenous administration of hAT-MSCs overexpressing IFN-b/LIF, reported a reduction in demyelination, an increase in the number of Olig2+ cells, and an elevation in the myelin basic protein (MBP) expression. This can, in turn, increase the production of myelin (Yousefi et al., 2022).

Due to their low immunogenicity and little to no ethical issues, MSC treatments for MS are also beginning to enter clinical trials. One of these includes a phase II double-blind trial on patients with active progressive MS, comprising 28 males and 20 females, conducted in Israel over a period of 14 months (Identifier: NCT02166021) (Petrou et al., 2020). This trial involved randomization of patients into three groups: intrathecal (IT) treatment, intravenous (IV) treatment or sham injections. Half of the patients belonging to each of the treatment groups received autologous BM-MSCs transplantation (1 × 10^6^/kg), followed by retreatment in 6 months, while the other half received sham injections during the 6-month follow-up. The patients that were initially assigned to the sham group were randomly divided into IT and IV-treatment groups at the 6-month follow-up and were administered with respective treatments. During the 1-year follow up, researchers noted that 58.6% of IT-treated and 40.6% of IV-treated patients had no indication of clinical symptoms, as opposed to only 9.7% of patients in the control group (Petrou et al., 2020). The MSC-IT group also scored significantly better on the timed 25-foot walking test, 9-hole peg test and cognitive tests. In addition, new results from this trial, published in early 2022., also demonstrate a significant decrease in cerebrospinal fluid (CSF) NF-L levels in 60% of patients that underwent MSC-IT treatment (*p* = 0.001) (Petrou et al., 2022). Interestingly, that effect was also observed, be it to a much lesser extent, in the MSC-IV treatment group, where it amounted to 33% of patients. As such, this trial has suggested that MSCs appear to be a viable treatment option for MS, with the most optimal delivery method being intrathecal application.

On the other hand, Uccelli et al. have performed a randomized, multi-center, double-blind, placebo-compared, cross-over phase I/II clinical trial with autologous bone-marrow derived MSCs—the MEsenchymal StEm cells for Multiple Sclerosis (MESEMS) study (Uccelli et al., 2019). As opposed to the classically-designed trial by Petrou et al., MESEMS was designed to merge partially independent clinical trials to overcome funding constraints. This clinical trial was conducted at 15 sites in nine countries (Italy, Canada, Austria, Denmark, France, Iran, Spain, Sweden, and the United Kingdom) from July 2012 until July 2019 (Uccelli et al., 2021). It included 144 individuals who were randomly allocated to receive IV infusion of autologous BM-MSCs (n = 69) or placebo (n = 75). At week 24 of the study, the MSC-treatment group received placebo, while the placebo group received a single IV dose of BM-MSCs. The follow-up was set at 48 weeks. Even though the study did meet the primary safety endpoint, wherein no serious adverse events were reported in the MSC group, it failed to meet the primary efficacy endpoint, set as “the number of gadolinium-enhancing lesions (GELs) counted over week 4, 12, and 24” (Uccelli et al., 2021).

Even though both studies used similar methods for BM-MSC delivery, with the MESEMS trial including three times more patients, there appears to be little consensus in whether BM-MSCs should be used to treat active MS. Despite the positive trend in the number of GELs reported by Petrou et al., the MESEMS trial demonstrated no significant differences between the treatment groups within a larger study sample. As such, further studies are needed to address the effects of MSCs on a variety of parameters as they relate to tissue repair. To optimize and standardize the administration protocol as well as facilitate easier comparison of obtained results, a multi-center trial approach should be utilized not only in phase III but also phase I/II clinical trials.

### 3.4 Glioblastoma multiforme

Glioblastoma multiforme (GBM) is an adult malignant primary tumor of the CNS. The mean survival time of patients with GBM is 12–14 months (Wen and Kesari, 2008). As GBM’s cancerogenesis remains unknown, recent decade has seen a rise in hypotheses underlying its etiology. These include the “clonal evolution model”, and the “cancer stem cell (CSC) hypothesis” (Rahman et al., 2011; Li et al., 2012a).

Like other tumors, GBM has the potential to attract resident MSCs (Yang et al., 2016), with GB-MSCs being the key component of the CSC niche (Tumangelova-Yuzeir et al., 2019). When exposed to such an environment, stem cells can undergo a process called “stromal corruption”, wherein the residing cells get modified by the tumor to favor its development (Fomchenko et al., 2011). Interestingly, there is a plethora of findings which indicate that, besides neural stem cells (NSCs), resident MSCs can also undergo the process of stromal corruption; namely, while some GB-MSCs are conventional bone-marrow derived MSCs, other have the distinctive genetic traits associated with CSCs (Hossain et al., 2015; Tumangelova-Yuzeir et al., 2019). A 2019. study by Tumangelova-Yuzeir et al. has shown that GB-MSCs express and secrete immunosuppressive molecules and factors, including IL-6, TGFβ, CCL-2, PGE2, and sVEGF (Tumangelova-Yuzeir et al., 2019). Since these can influence the activity of T-cell, resulting in a decrease in Th17 lymphocytes and an increase in Tregs *in vitro*, they represent another potential mechanism underlying immune suppression by GBM. The same study also found that GM-MSCs induce an overexpression of CD14 and CD68 as well as an underexpression of HLA-DR and CD80 by monocytes. As such, there is a plethora of evidence evoking caution in the therapeutic use of MSCs in treatment of GBM due to their critical involvement in the tumor microenvironment (TME), where they seem to actively participate in cancer development and progression (Bajetto et al., 2020).

Nevertheless, MSCs are still being researched as a potential therapeutic approach for a variety of CNS tumors, including GBM (Redjal et al., 2015; Jiang et al., 2016; Jiang et al., 2018). Here, MSCs present themselves as an interesting vehicle for delivery of microRNA (miR) with anti-cancer properties (Lee et al., 2013; Lang et al., 2018; Sharif et al., 2018). For example, a 2018. study by Lang et al. used lentiviral vectors to engineer BM-MSCs that produce EVs carrying high levels of miR-124a, an anti-glioma agent effective against glioma stem cell (GSC) lines (Lang et al., 2018). Following *in vitro* treatment of GSCs with exosomes carrying miR-124a, the GSCs exhibited a significant reduction in viability and clonogenicity, when compared with the control. Additionally, subsequent *in vivo* treatment of mice with intercranial GSC267, accompanied by a systemic delivery of exosomes containing miR-124a, led to a long-term survival of 50%, with no presence of tumor in histological analysis. Next, a 2016 study by Jiang et al. has shown that MSCs engineered to express the tumor necrosis factor-related apoptosis-inducing ligand (TRAIL) inhibit the growth of GBM, induce apoptosis and, in turn, extend animal survival (Jiang et al., 2016). A concise overview of *in vitro* and *in vivo* studies exploring the effects of MSCs application in glioblastoma multiforme can be found in Table 4.

**TABLE 4:** Summary of *in vitro* and *in vivo* studies investigating the beneficial effects of MSCs in glioblastoma multiforme.

Treatment	Disease model	Results	Mechanisms of action	Authors
CXCR4-overexpressing hAT-MSCs	GBM spheroids grown in an extracellular matrix	Decrease in tumor hypoxia	Enhanced long-term migration towards GBM	Jiang et al. (2018)
			Preferential penetration of the hypoxic tumor core	
TRAIL-overexpressing hAT-MSCs	Patient-derived GBM orthotropic xenografts in mice	Significant inhibition of tumor growth	Long-range directional migration towards GBM	Jiang et al. (2016)
		Extension of animal survival	Induction of rapid cell apoptosis	
hWJ-MSCs transfected with miR-124	U87 GBM cells	Enhanced chemosensitivity of GBM cells to temozolomide	Decrease in luciferase activity of the target gene CDK6	Sharif et al. (2018)
		Decreased migration of GBM cells		
BM-MSC-derived EVs carrying high levels of miR-124a	Glioma stem cell lines (GSC267, GSC20, GSC6-27, GSC8-11, GSC2-14)	Significant reduction in viability and clonogenicity of GSCs	miR-124a inhibition of GCS viability through knockdown of FOXA2	Lang et al. (2018)
BM-, AT-, P- and UC-MSCs transfected with miR-124 and miR-145 mimics	U87-derived xenografts in mice	Decreased migration of glioma cells	Attenuation of luciferase activity of the reporter target genes, SCP-1 and Sox2	Lee et al. (2013)
		Decreased self-renewal of GSCs		
GFP-labeled commercial hMSCs carrying Delta-24-RGD	Mice harboring orthotopic U87MG or U251-V121 xenografts	Inhibition of glioma growth	Localization of hMSCs-Delta24 to gliomas, probably *via* the tumor vasculature	Yong et al. (2009)
		Extension of animal survival		

Some of these strategies have been further extended into clinics, wherein there currently exist at least two active clinical trials evaluating the prospects of MSC-based treatments of GBM (Calinescu et al., 2021). The first one, being executed at the M.D. Anderson Cancer Center in Texas (United States), is building upon their results in preclinical models of GBM, wherein allogenic MSCs loaded with OVs (oncolytic viruses) were administered into the carotid artery and showed promising results (Identifier: NCT03896568) (Yong et al., 2009). This phase I, open-label clinical trial uses the conditionally replicating oncolytic adenovirus Delta24-RGD to take advantage of the tumor-tropism of the MSCs, potentially limiting the spread of the virus to other organs. This also allows MSCs to cross the BBB and distribute widely in the tumor (Identifier: NCT03896568). Another study, conducted at the CHA University in South Korea, is using MSCs that express the suicide gene cytosine deaminase (CD) and transplanting them into patients with recurrent glioblastoma (Identifier: NCT04657315). This is an open-labeled, phase I/II clinical trial for evaluation of maximum tolerated dose, safety, and efficiency.

### 3.5 Ischemic stroke

Ischemic stroke (IS) is a pathological condition caused by an interruption in the blood circulation through the brain (Thayabaranathan et al., 2022). It represents the most common type of stroke and leads to neuronal cell death. Nevertheless, current treatments for IS, which include thrombolysis and mechanical thrombectomy, have an extremely narrow therapeutic window and, as such, bring benefits for only 5% of patients (Nogueira et al., 2018). Since ischemic stroke includes many pathophysiological events which occur in parallel, from ion imbalance and hyperinflammation to activation of various types of cell death, transplantation of stem cells might bring a multitude of benefits (Kuriakose and Xiao, 2020).

Among numerous published articles which report positive effects of MSC transplantation in animal models of stroke, many of them were performed in the last decade (Xia et al., 2020; Xu et al., 2020; Li et al., 2021b; Zhuo et al., 2021) (Table 5). For example, stroke rats that have been administered with hUC-MSCs displayed a substantial increase in motor function, coupled with elevated metabolic activity and enhanced vascularization within the infarct cortex (Lin et al., 2011). There was also a trend toward reduction in the infarct volume. Similarly, Zhuo et al. performed both an *in vitro* experiment on an oxygen-glucose deprivation/reperfusion (OGD/R) neuron model, as well as an *in vivo* experiment on MCAO rats using the ischemic-hypoxic, preconditioned, olfactory mucosa MSCs (IhOM-MSCs) (Zhuo et al., 2021). Their results demonstrated that IhOM-MSCs protect mitochondrial function and inhibit cell death. On the other hand, intranasal delivery of BM-MSCs was tested in day 7, postnatal rat pups affected by stroke (Wei et al., 2015). Authors reported a decrease in ischemic cortex volume, reduction in BBB disruption, as well as an increase in angiogenesis, neurogenesis, and cerebral blood flow. Besides 2D cell cultures, some researchers are also looking into transplantation of 3D-cultured MSCs (Li et al., 2021b). When compared to 2D-cultured MSCs, transplantation of 3D-cultured MSCs dramatically decreased the infarct volume and resulted in an enhanced cell engraftment into the ischemic region. Additionally, Li et al. also reported decreased levels of proinflammatory cytokines and suppressed microglial activation in the 3D treatment group (Li et al., 2021b). Another interesting finding was achieved after intravenous transplantation of BM-MSCs, which resulted in a reduced loss of neurons in the hippocampus (Acosta et al., 2015).

**TABLE 5:** Summary of *in vitro* and *in vivo* studies investigating the beneficial effects of MSCs in ischemic stroke.

Treatment	Disease model	Results	Mechanisms of action	Authors
hUC-MSCs	MCAO rat model	Increase in motor function	Release of neuroprotective ad growth-associated cytokines (BDNF, bFGF, CXCL-16, VEGFR-3, and angiopoietin-2)	Lin et al. (2011)
		Elevated metabolic activity		
		Enhanced vascularization of the infarct cortex		
Ischemic-hypoxically preconditioned OM-MSCs	OGD/R neuron model	Protection of mitochondrial function	Upregulation of the downstream target genes GRP78 and Bcl-2 by miR-181a	Zhuo et al. (2021)
	MCAO rat model	Inhibition of apoptosis and pyroptosis of neurons		
Hypoxically preconditioned hBM-MSCs	MCAO rat model	Improved sensorimotor and olfactory functional recovery	Increase in angiogenesis, neurogenesis, and cerebral blood flow	Wei et al. (2015)
		Improved social behavior	Decrease in ischemic cortex volume	
			Reduction in BBB disruption	
hBM-MSCs	MCAO rat model	Reduction in striatal infarct and peri-infarct area	Not specified	Acosta et al. (2015)
		No change in the rate of hippocampal cell loss		
		Amelioration of stroke-induced neuroinflammation		
BM-MSC-derived exosomes containing miR-138-5p	OGD/R neuron model	Reduction in neurological impairment	Protection of astrocytes *via* downregulation of miR-138-5p target gene LCN2	Deng et al. (2019)
	MCAO mouse model	Inhibition of astrocytes’ inflammatory response		
BM-MSC-derived exosomes containing miR-133-b	MCAO rat model; astrocyte/neuron-MSC cocultures	Significant increase in neurite branch number and total neuron length in treated neurons	Regulation of neurite outgrowth by transfer of miR-133b to neural cells *via* exosomes	Xin et al. (2012)
BM-MSC-derived exosomes containing miR-133-b	MCAO rat model	Significantly improved functional recovery	miR-133b transfer to astrocytes and neurons, regulating their gene expression	Xin et al. (2013)
		Enhanced axonal plasticity and neurite remodeling in the ischemic boundary zone at day 14 after MCAO		
MVs derived from hAT-MSCs treated with normal or stroke-injured rat brain extract	pMCAO rat model	Reduction in inflammation	Attenuation of TNF and progranulin (PGRN) expression	Lee et al. (2016)
		Enhanced neurogenesis and increased endogenous neurogenesis	Upregulation of angiogenesis-, neurogenesis-, and apoptosis-related genes	

On top of transplantation of MSCs into the affected tissue, be it with direct or systemic delivery, researchers are also exploring the use of EVs and miRNAs for treatment of a variety of ischemic diseases. For example, a 2019. study by Deng et al. has shown that MSC-derived EVs loaded with miR-138-5p are successful in preventing additional astrocyte damage caused by oxygen/glucose deprivation (OGD) following endocytosis in mice with MCAO (Deng et al., 2019). The results of this study demonstrated reduced neurological impairment following treatment. Similarly, the application of miR-133b-enriched MSC-derived EVs in MCAO rats promoted neural plasticity and enhanced neurite outgrowth (Xin et al., 2012; Xin et al., 2013). Others have used MSC-derived microvesicles (MVs) pretreated with normal rat brain extract (NBE-MSC-MVs) or stroke-injured rat brain extract (SBE-MSC-MVs) to investigate their impact on ischemic brain damage caused by permanent MCAO (pMCAO) (Lee et al., 2016). Interestingly, this study found that transplantation of pretreated MSC-MVs had a significantly greater efficacy in ameliorating ischemic injury and improving functional recovery than that of MSC-MVs. Additionally, MSC-MVs that have been treated with the brain-extracts have been shown to decrease the inflammatory response, boost neovascularization, and enhance endogenous neurogenesis (Lee et al., 2016). Likewise, intravenous application of MSC-derived EVs also resulted in neurological functional recovery as well as increased angiogenesis and neurogenesis in MCAO mice (Xu et al., 2020).

After confirming that transplantation of stem cells in patients affected by stroke is a safe and well-tolerated procedure (Qiao et al., 2014), more advanced stage clinical trials aimed to answer the question if stem cells could bring measurable benefits. One of these is a prospective, open-label randomized phase III clinical trial performed on 54 patients with severe middle cerebral artery territory infarct (Identifier: NCT01716481) (Lee et al., 2022). This trial included transplantation of autologous BM-MSCs and reported improvements in motor function in the intravenously treated group in 90 days after the treatment. Additional research of patient’s biomarkers also demonstrated a significant increase in numbers of circulating EVs in stroke patients treated with BM-MSCs (*p* = 0.001), with an increase in miRNAs related to neurogenesis and neuroplasticity (e.g., miRNA-18-a-5p) (*p* = 0.034) (Bang et al., 2022). Likewise, improvement in motor function was also reported by a different clinical trial, this time a phase II, single-center, open-label RCT, with a 2-year follow-up (Identifier: NCT00875654) (Jaillard et al., 2020). On the other hand, another similar trial did not report any neurological recovery or functional outcome improvement at 12 months, but rather only a reduction in the volume of infarcted tissue (Identifier: NCT01461720) (Law et al., 2021).

On top of BM-MSCs, AT-MSCs are also being used in clinical trials for treatment of acute ischemic stroke. A phase II, randomized, double-blind, placebo-controlled, single center pilot clinical trial was performed on 13 patients (4 receiving AT-MSCs and 9 placebo) over 60 years of age with a moderate to severe acute ischemic stroke (Identifier: NCT04280003) (de Celis-Ruiz et al., 2022). The patients received IV infusion of AT-MSCs within the first 2 weeks of stroke and, at 24-month follow-up, showed a non-significantly lower median National Institutes of Health Stroke Scale (NIHSS) score, when compared to the placebo group (interquartile range, 3 [3–5.5] vs. 7 [0–8]).

## 4 Challenges for MSC-based therapies of the nervous system

### 4.1 Immunocompatibility

Interestingly, MSCs can exhibit both immunosuppressive and proinflammatory activities. These effects depend on the cell’s level of stimulation by inflammatory cytokines, chemokines (including PGE2, TGF-B, IL-6, IL-10, HLAG5), metalloproteinases, indoleamine-2,3-dioxygenase (IDO1), and nitric oxide (NO). MSCs immunosuppressive activity can, therefore, be utilized as a form of prevention of both allograft rejection episodes and an abnormal autoimmune or inflammatory response (De Miguel M et al., 2012). On a molecular level, it is known that MSCs have immunosuppressive effects in the presence of NO (Ren et al., 2008; Han et al., 2012). Contrastingly, in areas which lack NO, namely, those regions where the inducible nitric oxide synthase (iNOS) is inhibited, MSCs enhance the proliferation of the immune cells. Furthermore, Qin et al. have discovered that, in the presence of the NOS inhibitor L-NMMA, MSCs are not successful in inhibiting the proliferation of T-cell in rat models (Qin et al., 2021). These findings clearly imply that the production of NO, or an increase NOS2 activity, is required for the onset of MSC-mediated immunosuppression. Like NO, indoleamine 2,3-dioxygenase also acts as a switch in MSC- mediated immunomodulation (Plumas et al., 2005; Krampera et al., 2006).

While some research suggests that MSCs may contribute to cancer development, other indicates that they have suppressive effects on tumor development (Li et al., 2012b). The mechanisms underlying these suppressive effects are explained by inhibition of proliferation-related signaling pathways PI3K/AKT, induction of cell cycle arrest and, subsequently, the reduction of cancer growth (Lu et al., 2019). Contrastingly, other studies have shown that MSCs can undergo differentiation into cancer-associated fibroblasts (CAFs) and, thereby, actively promote cancer progression (Jotzu et al., 2011; Barcellos-de-Souza et al., 2016; Aoto et al., 2018).

### 4.2 Stemness stability and differentiation of MSCs

The stemness properties of MSCs are affected by a variety of factors, including the isolation method, individual variability of the source tissue, health of the donor and the history of cell culture in question (Mastrolia et al., 2019). Dental pulp mesenchymal stem cells (DP-MSCs) represent 10% of dental pulp cells. Even though they exhibit higher proliferation rates, DP-MSCs produce lower quantities of proangiogenic factors than BM‐MSCs and AT‐MSCs *in vitro* (d’Aquino et al., 2007). Nevertheless, other studies have shown that chemokines and neurotrophins released by DP-MSCs play a key role in neuroprotection and response to injuries of the nervous system (Mead et al., 2014; Yu et al., 2016).

Interestingly, MSCs also exhibit donor‐related variations. These can be due to the patients’ gender, BMI, donor site, age, and underlying diseases (Mastrolia et al., 2019). A 2016. study by Sammour et al. has shown that MSCs isolated from female donors are more efficient in reducing lung inflammation in a rat model, when compared with male MSCs (Sammour et al., 2016). On the other hand, the hormonal variations women are exposed to, especially during menopause, decrease the osteogenic potential of resident stem cells (Zhu et al., 2009). Furthermore, Ogawa et al. also verified the existence of gender variability in AT-MSCs by finding a higher amount of Peroxisome Proliferator Activated Receptor‐ϒ2 (PPAR‐ϒ2), an adipogenesis marker, in cells derived from female mice (Ogawa et al., 2004; Zhu et al., 2009; Sammour et al., 2016).

Some studies have also reported age‐related changes in MSCs (Alonso-Goulart et al., 2018; Mahmood et al., 2018; Mohamed-Ahmed et al., 2018). For example, MSCs from elderly people have lower superoxide dismutase activity, and higher levels of reactive nitrogen and reactive oxygen species (RNS and ROS, respectively) (Stolzing et al., 2008). This results in oxidative damage to MSCs and, subsequently, apoptosis. Additionally, p53 and p21, which are recognized for their pro-apoptotic activity, are both upregulated in aged MSCs, while the expression of Notch1 receptor, which is implicated in bone development, is downregulated (Stolzing et al., 2008).

For MSC-based therapies to reach significant levels of success in treatment of neurological disorders, they must also account for the inherent influence that the diseases have on the cell’s regenerative capacities. For example, Diabetes Mellitus (type 2 diabetes) has a negative impact on MSCs function, reducing their ability to produce new blood vessels by downregulating pro-angiogenic factors (Rezabakhsh et al., 2017). Moreover, BM‐MSCs isolated from diabetic patients exhibit decreased paracrine secretion and a greater proclivity to develop into adipocytes (Ferland-McCollough et al., 2018).

BMI has also been reported to influence the differentiation and proliferation abilities of adipocytes (Geissler et al., 2014). As such, overweight individuals boast compromised cell differentiation, proliferation, and DNA telomere length. This is accompanied by cells’ diminished potential for self‐renewal and early onset of apoptosis. Additionally, high BMI also has a detrimental effect on both AT- and BM-MSCs, as evident in substantially degraded osteogenic and attenuated adipogenic differentiation, impaired cell proliferation, and enhanced senescence (Ulum et al., 2018). It is interesting to note that, following significant weight reduction, DNA damage is decreased, and cell viability and replicative longevity are both improved (Mitterberger et al., 2014).

Donor-related variations in MSCs are also visible in menstrual-blood-derived MSCs (MB-MSCs). For example, MB-MSCs from women with endometriosis (eMB-MSCs) are morphologically different from healthy patient‐derived MSCs (Nikoo et al., 2014). They are characterized by higher proliferation and invasion potentials as well as the ability to manipulate inflammatory responses to their advantage, which further propagates the development of endometriosis.

Finally, MSCs properties are also impacted by some pharmacological agents and treatment methods, including immunosuppressive drugs (Tsuji et al., 2015), antitumor drugs (Pike et al., 2015), and radiotherapy (Poglio et al., 2009). Similar to these, prolonged use of morphine impairs angiogenesis and endothelial progenitor cell activation (Holan et al., 2018). It also exerts a deleterious effect on the proliferation and differentiation of MSCs, altering their secretory capabilities and hindering wound repair (Holan et al., 2018).

### 4.3 Heterogeneity

Even though a multitude of studies have revealed the fascinating benefits of MSCs in tissue repair, making them an attractive research target in the field of regenerative medicine, MSCs from different sources exhibit significantly different properties (Andrzejewska et al., 2019). For example, MSCs of fetal origin differ from cells obtained from adult tissues in that they proliferate more quickly and can undergo more *in vitro* passages before senescence. (Hass et al., 2011). On the other hand, adult-isolated BM- MSCs and AT-MSCs possess a higher degree of stemness, reflected in their ability to create a larger number of fibroblast colonies (CFU-F) (Kern et al., 2006; Heo et al., 2016).

Interestingly, even MSCs obtained from individual donors can exhibit some clear differences. Among BM-MSCs isolated from donors of different ages and sexes, studies found significant differences in their proliferation rates, osteogenesis, and the level of bone remodeling marker (alkaline phosphatase, ALP) activity (Phinney et al., 1999). Interestingly, no correlation between these and the sex or the age of the donors was said to be found. Yet, other studies have shown that the properties of BM-MSCs heavily depend on the donor’s age. For example, cells isolated from older individuals exhibited increased apoptosis and diminished proliferation, as well as a decreased capacity for differentiation toward osteoblasts (Zhou et al., 2008). Heo et al. also demonstrated that there is considerable interdonor variation in distal-less homeobox 5 (DLX5) gene expression between MSCs derived from different tissues (Heo et al., 2016).

In order to facilitate easier recognition of specific molecular and functional phenotypes, which appear to be related to harvesting techniques and tissue sources (Walter et al., 2020), Colter et al. have categorized MSCs into three subpopulations, based on their morphology: “spindle-shaped proliferating cells resembling fibroblasts” (type I); “large, flat cells with a clearly marked cytoskeleton structure containing a number of granules” (type II); and “small, round cells with high self-renewal capacity” (type III) (Colter et al., 2000).

### 4.4 Adverse effects

Despite the beneficial outcomes that MSC treatment promises, there are many challenges and controversies relating to MSCs’ application into the human cell niche that require more research. With that, some of the main potential risks pertaining to MSCs therapy include: 1) potential differentiation into undesirable cell types and pro-tumorigenic activity, 2) uncontrolled immune response, 3) short survival after implantation, 4) insufficiently researched differentiation capacities, and 5) unspecified optimal doses and route of cell administration.

MSCs may exhibit pro-tumorous activity by enhancing tumor invasion through secretion of CCL5 (Karnoub et al., 2007) as well as inhibiting apoptosis through secretion of pro-survival factors VEGF and bFGF (König et al., 1997; Dias et al., 2002; Zhu et al., 2017). As such, since MSCs can exhibit both immunosuppressive and immunomodulatory effects, their administration can result in an uncontrolled immune response on either the global or the local level (Rivera-Cruz et al., 2017).

When discussing MSCs short survival following implantation, several studies have demonstrated the occurrence of massive death of MSCs shortly after transplantation through activation of hypoxia signaling pathways and Caspase 3-mediated apoptosis (Preda et al., 2021). Interestingly, a study by Deschepper et al. has shown that the massive death of MSCs at day 6 was induced by ischemic conditions (low pO_2_ and glucose depletion), while cells in hypoxic conditions (low O_2_) remained viable until day 12 (Deschepper et al., 2011).

Despite all benefits pertaining to future treatment strategies, some questions about the differentiation capacity and the use of MSCs remain. These include the exact mechanism of their action, their safety in routine clinical use, and their migration patterns within the tissue (Musiał-Wysocka et al., 2019). As such, much of the research within the field suggests that, for maximum treatment effectiveness, different clinical indications and diseases necessitate alternative administration methods (Galipeau and Sensébé, 2018; Caplan et al., 2019). Most prominent routes of cell administration include: 1) systemic delivery with intravenous (IV) and intraatrial (IA) delivery, and 2) local delivery with topical, intramuscular, direct tissue injection and transepi- or endo-cardial applications. Still, neither of these routes is without its faults; wherein both IV and IA delivery pose risks for stroke through formation of emboli or thrombi. Additionally, and when it comes to systemic administration, the cells within the circulation are exposed to innate host immune cells, potentially resulting in unwanted effects. On the other hand, direct injection can induce yet-undefined cell to cell interactions and potentially activate other secondary signaling systems (Caplan et al., 2019; Lukomska et al., 2019).

Other questions that arise in the debate on whether MSCs treatment should be widely applicable include the efficiency of MSCs isolation from elderly patients and patients with systemic diseases such as diabetes, rheumatoid arthritis, and inflammatory diseases, since their cells can be affected by the disease. The element of low efficiency of MSCs that have been isolated from elderly patients could be circumvented through banking of MSCs at a younger age (Kokai et al., 2017). On top of these, obesity and BMI have also been shown to determine the fate, and reduce the efficiency, of MSCs (Pachón-Peña et al., 2016; Dufrane, 2017; Liu et al., 2017). To limit the risk of adverse effects, novel techniques for MSC isolation and *ex vivo* processing for clinical use should be developed. (Lukomska et al., 2019).

### 4.5 Tumor-promoting ability

**FIGURE 3:**
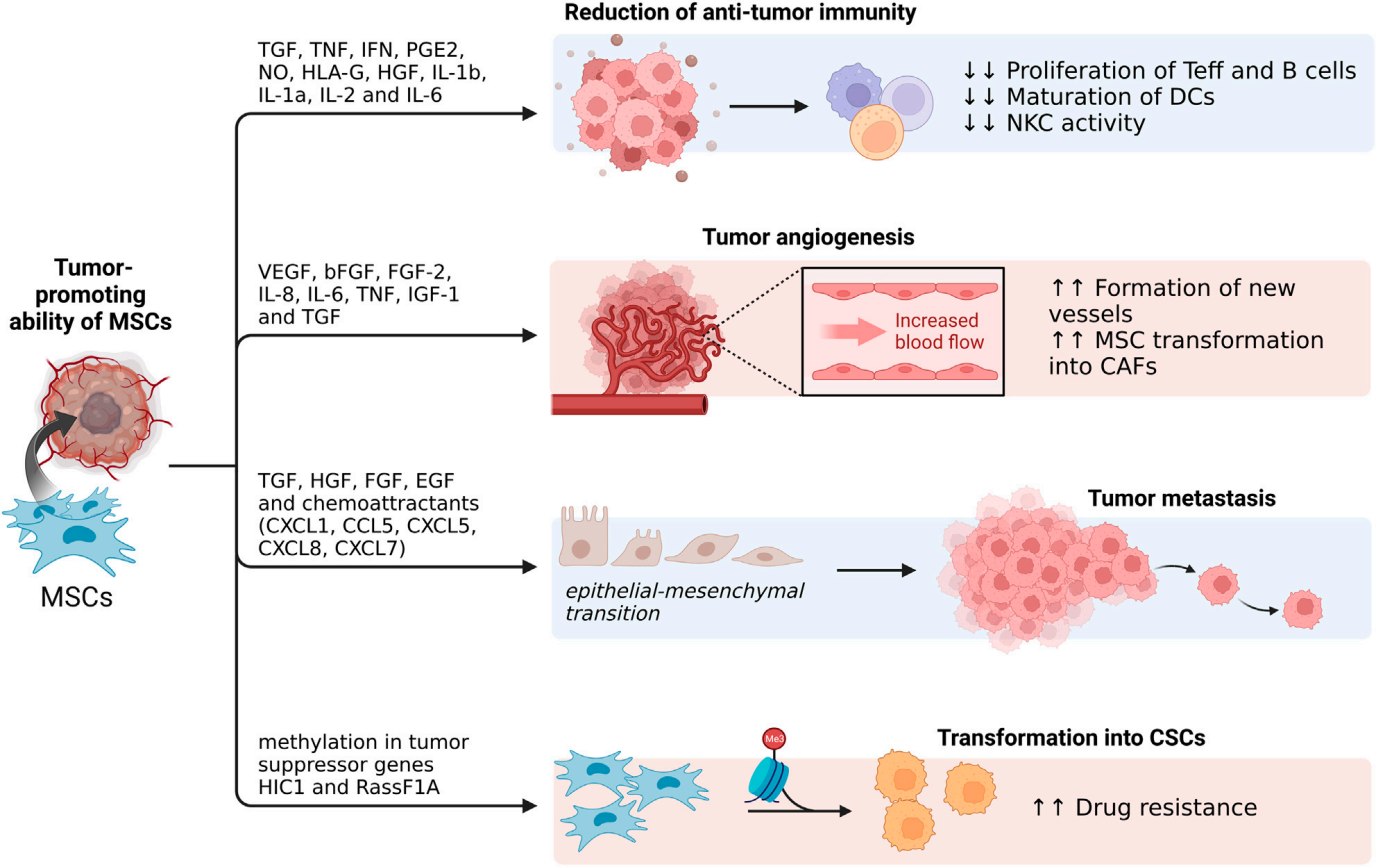
Tumor-promoting ability of MSCs. *On top of playing an important role in reduction of anti-tumor immunity, MSCs can undergo transformation into CSCs as well as promote epithelial-mesenchymal transitions, leading to tumor metastasis. Additionally, through secretion of various factors, MSCs can also play a role in tumor angiogenesis (Created with* *BioRender.com* *)*.

Even though MSCs show the capacity of navigating towards tumor sites, many studies also warn of their protumor activity, namely, 1) immunosuppression (Jiang et al., 2005; Sato et al., 2007; Ren et al., 2008; Volarevic et al., 2010; Lee et al., 2015), 2) promotion of angiogenesis (Birnbaum et al., 2007; Zacharek et al., 2007; Huang et al., 2013; O’Malley et al., 2016), 3) transition to cancer-associated fibroblasts (Spaeth et al., 2009; Barcellos-de-Souza et al., 2016; Ishihara et al., 2017), 4) inhibition of apoptosis in cancer cells (Hung et al., 2007a; Hung et al., 2007b; Sanchez et al., 2009; Efimenko et al., 2011), 5) increase in metastatic ability and tumor growth (Zhu et al., 2006; ting et al., 2009), 6) induction of epithelial-mesenchymal transition (EMT) (Martin et al., 2010; Thomas and Karnoub, 2013; Xue et al., 2015; Esposito et al., 2019; Yan et al., 2021; Yin et al., 2022), and 7) promotion of drug resistance (Scherzed et al., 2011; Ji et al., 2015; He et al., 2019; Han et al., 2021; Shi et al., 2021) (Figure 3). Although the aforementioned studies observed and quantified the effects of resident MSCs on tumor progression and other associated activities, MSCs tumor-promoting ability is worth keeping in mind when designing any new therapeutic approaches based on this cell type (Liang et al., 2021).

When discussing MSCs role in suppressing both the innate and adaptive immune response, they accomplish this through secretion of TGF α , TNF β , IFN γ , PGE2, NO, HLA-G, HGF, IL-1b, IL-1a, IL-2 and IL-6 (Rivera-Cruz et al., 2017; Liang et al., 2021). In turn, these factors reduce proliferation of effector T-cell and B-cell, maturation of dendritic cells (DCs), natural killer cell (NKC) activity and secretion of IgG. This all results in a reduction in anti-tumor immunity.

MSCs have also been shown to promote tumor angiogenesis through release of VEGF, bFGF, FGF-2, IL-8, IL-6, TNF, IGF-1 and TGF β as well as subsequent transformation into smooth muscle cells (Gu et al., 2018) and pericytes, coupled with formation of new tumor vessels (Crisostomo et al., 2008; Efimenko et al., 2011; Huang et al., 2013; Kalluri, 2016; Liang et al., 2021). On top of transforming into smooth muscle cells, MSCs can also undergo conversion into cancer-associated fibroblasts (CAFs) (Barcellos-de-Souza et al., 2016; Ishihara et al., 2017). On the other hand, promotion of tumor metastasis often follows the epithelial-mesenchymal transition induced by MSCs secretion of TGF β , HGF, FGF and EGF (McAndrews et al., 2015; Berger et al., 2016; Hill et al., 2017). MSCs can also secrete chemoattractants, including CXCL1, CCL5, CXCL5, CXCL8 and CXCL7, which promote tumor cell migration to metastatic lesions (McAndrews et al., 2015). Finally, some studies have shown that resident MSCs promote drug resistance. This property is particularly prominent is CSCs, or cancer stem cells.

CSCs are cancer cells that have undergone EMT (Mani et al., 2008). Not only can MSCs support the CSCs microenvironment, but they can also transform into CSCs themselves. They can accomplish this through methylation in the tumor suppressor genes HIC1, and RassF1A, occurrence which was identified in some cases of advanced ovarian cancer (Teodoridis et al., 2005). This, in turn, increases the tumor’s resistance to cisplatin and increases the risk of tumor reoccurrence (Liang et al., 2021). Even though cisplatin administration caused cell death in the control group of BM-MSCs, Teng et al. found that this treatment was substantially less effective in transfected BM-MSCs. (Teng et al., 2011).

### 4.6 Technical and societal challenges

To prevent cell contamination and facilitate the application of a highly standardized protocol, the use of MSCs for therapeutic purposes requires highly skilled professionals. Even though many clinical trials are underway, we still lack a standardized protocol outlining the appropriate steps for isolation and maintenance of MSC cultures (Loo and Wong, 2021). The importance of such a standardized protocol lies in the fact that it can facilitate easier one-to-one comparison of varied experimental studies and clinical trials for the purpose of determining the most optimal delivery method and concentration.

With an increased and widespread public interest in stem cell therapy, recent years have also seen a rise in biobanking. Although such facilities provide their users with access to highly multipotent stem cells at will, and when in need, they are also at risk of exploitation (Loo and Wong, 2021). This is particularly prominent in privately-operated biobanks, which are at risk of breaches of privacy as well as commercialization of health data (Winickoff, 2015).

Additionally, people that are using these biobanking services appear to fall into a specific social group—white, middle-class, and well-educated individuals. This results in an unintentional exclusion of individuals not fitting into these norms, namely, those that are indigenous, lower-class, and from culturally diverse communities (Prictor et al., 2018). This prejudice is also reflected in the recruitment process wherein, for example, the participants in the UK Biobank were more likely to be women, in better health and living in wealthier areas (Fry et al., 2017). Similar trend was also noted in the Estonian Biobank, which was biased towards women and younger people, with underrepresentation of some minorities (Leitsalu et al., 2015). Not only is this bias limiting marginalized individual’s access to biobanks, but it is also negatively impacting scientific enquiry since a wide variety of research employs biobank samples and data (Prictor et al., 2018). Still, with awareness comes the ability to rectify these issues of inclusivity and representativeness—something we must actively strive towards in the upcoming years.

## 5 Prospects for MSC-based therapies

### 5.1 Novel biomaterials

Even though a variety of bioengineering approaches aiming to facilitate and optimize MSC treatments have emerged throughout the decade, ensuring efficient engraftment and survival of implanted MSCs remains a challenge (Hmadcha et al., 2020b). To address this obstacle, researchers are using biomaterials as scaffolds designed to improve the retention of transplanted cells (Zeng et al., 2015b; Zhang et al., 2018b; Ryu et al., 2019c; Yan et al., 2019; Deng et al., 2020; Sahab Negah et al., 2020; Ma et al., 2021b; Restan Perez et al., 2021). Efforts are being made to produce ideal biodegradable and biocompatible scaffolds based on both synthetic and natural polymers that mirror the biological and mechanical properties of the target tissue (Doblado et al., 2021). As such, on top of facilitating MSCs proliferation and survival, this material should also provide a suitable environment for neuronal survival, facilitate axonal extension and projection as well as mimic the tissue biomechanics (Vijayavenkataraman, 2020).

A study by Negah et al. utilized an experimental TBI model in rats to assess whether RADA4GGSIKVAV (R-GSIK), a self-assembling nano-peptide scaffold, brings quantifiable benefits in the form of functional improvement and decrease in neuroinflammation (Sahab Negah et al., 2020). Interestingly, a significant functional recovery was observed in rats that received MSCs with R-GSIK scaffolding, as opposed to those in the control group. Additional analysis revealed that both MSCs- and MSCs + R-GSIK- treated groups contain smaller numbers of reactive astrocytes and microglia, accompanied by a considerable decrease in proinflammatory cytokines such as TLR4, TNF and IL6 (Sahab Negah et al., 2020). On the other hand, a study by Yan et al. used the “300 g weight free fall impact” traumatic brain injury model to test the effectiveness of BM-MSCs on a collagen-chitosan scaffold (Yan et al., 2019). They reported that the transplantation of BM-MSCs and collagen-chitosan scaffolds reduced the modified neurological severity scores and diminished neurodegeneration. Interestingly, the therapeutic effects of BM-MSCs that have been combined with the scaffold were superior to stereotaxic injection of BM-MSCs alone. All in all, these studies indicate that transplantation of MSCs embedded within various scaffolds can promote the recovery of neuropathological injury and, as such, is of great significance in advancing our approaches towards treating traumatic injuries of the nervous tissue.

While most of these studies examined the efficiency of this novel MSC delivery method on traumatic brain or spinal cord injuries, a study by Ryu et al. quantified the effectiveness of allogeneic AT-MSC sheets in treatment of MCAO (Ryu et al., 2019c). The study included 10 animals in the control, and 10 in the treatment group. They were observed over a period of 14 days after transplantation. The results of the study demonstrate an increase in functional angiogenesis and neurogenesis in the treatment group, followed by behavioral improvements.

Furthermore, Zhang et al. have created an injectable hydrogel made of sodium alginate (SA) and hyaluronic acid (HA) to act as a tissue scaffold, supplying the stem cells with a more favorable microenvironment after implantation (Zhang et al., 2018b). This HA/SA hydrogel is characterized by porous structures that appear to be appropriate for stem cell loading and exhibit good rheological behavior. Following the loading of hUC-MSCs into the hydrogel *in vitro*, the cells exhibited higher viability and proliferation as compared to the control group. Interestingly, the HA/SA scaffold also contributed to the regeneration of endogenous neural cells.

Since development of most novel biomaterials meant to facilitate improved cell retention and viability following transplantation is still underway, there is only a limited number of solutions that have reached the phase of clinical trials. Nevertheless, one small clinical trial, evaluating the efficiency of a collagen scaffold has recently been completed. A phase I clinical trial reported by Deng et al., performed at the Characteristic Medical Center of Chinese People’s Armed Police Force, used collagen scaffolds loaded with hUC-MSCs for treatment of acute complete spinal cord injury (Deng et al., 2020). The trial comprised 20 patients in the treatment, and 20 in the control group. The collected evidence suggests that the transplantation of hUC-MSCs onto a collagen scaffold can increase patient’s functional recovery, albeit the findings must be validated in a multicenter clinical study with a greater number of participants (Deng et al., 2020).

Even though many of the studies pointed towards clear advantages in using novel scaffolds to facilitate higher cell retention and survival, most natural biomaterials still exhibit very specific disadvantages which must be addressed prior to translation into clinical practice. These include: 1) batch variability, 2) short degradation period, 3) difficulty in purification, and 4) quality control (Xu et al., 2019b). Even though the structure of synthetic biomaterials can be controlled and, as such, can address some of the shortcomings of natural biomaterials, they remain a less optimal choice. The reason for that is their lack of cell adhesion properties and biological signals, making them less likely to direct cell fate on their own (Xu et al., 2019b). Additionally, biocompatibility and bioresorbability of these materials is often brought into question.

### 5.2 Electromagnetic fields

Even though freshly isolated MSCs have numerous positive attributes, they are extremely heterogeneous and come in limited quantities, owing to the patients age and gender (Hamid et al., 2022). As such, the development of novel methods for cell expansion is a critical step towards translation into clinics. Yet, classical methods of expansion of isolated cells in the form of extensive *in vitro* passages can result in cells with morphological, phenotypic, and genetic changes (Gharibi and Hughes, 2012). Even though considerable efforts have been made to facilitate expansion and enhance the proliferation of MSCs *in vitro*, without modifying their properties, most of these are still insufficient (Gharibi and Hughes, 2012). In this context, researchers are turning to the use of electromagnetic fields (EMFs), which have been shown not only to promote the proliferation of MSCs (Sun et al., 2009; Li et al., 2012c; Lim et al., 2013; Parate et al., 2020; Alipour et al., 2022) but also drive neurogenesis (Kim et al., 2013; Seong et al., 2014). One of the mechanisms behind this action was suggested to be the upregulation of Ca(v)1-channel expression and function (Piacentini et al., 2008). As expected, these effects are closely related to the frequency of applied EMFs, duration of treatment, and the differentiation stage of MSCs.

For example, a 2022. study by Huang et al. has shown that a concurrent transplantation of BM-MSCs and an application of pulsating electromagnetic fields (PEMFs) increases the expression of brain-derived neurotrophic factor (BDNF), nerve growth factor (NGF), and VEGF in C57BL/6 mice with T10 contusion injury (Huang et al., 2022). Following local BM-MSC transplantation into the injury site, the mice were exposed to PEMF (50 Hz, 1 mT, 1 h/day) for 8 weeks after the injury. The synergistic effect of PEMFs and BM-MSCs enhanced locomotor functional recovery of the affected animals, as seen in their behavioral assessment scores (Basso Mouse Scale, BMS). The animals within the treatment group also exhibited some signs of active preservation of neurons and an elevated rate of axonal growth visible in the expression of NeuN and MBP, respectively.

On the other hand, Feng et al. have used a combination of TMS and BM-MSCs to treat rat spinal cord injury (Feng et al., 2021). The study utilized 8-week-old female Sprague Dawley rats and established a model of SCI using the „weight-drop method“. This was followed by either monotherapy (TMS, BM-MSCs or PLX4720, RafI inhibitor) or combination therapy (TMS + BM-MSCs or TMS + PLX4720) (Feng et al., 2021). Even though the results demonstrated that monotherapy suppressed neuronal apoptosis and promoted axonal regeneration, these effects were much more prominent in the groups receiving one of the concurrent therapies. Most strikingly, combination treatment significantly reduced lesions within the spinal cord, decreased the rate of neuronal apoptosis and elevated the expression of NGF and BDNF (FENG et al., 2021).

Other researchers have demonstrated that pre-treatment of BM-MSCs with low intensity pulsed ultrasound (LIPUS) boosted the positive effects of BM-MSCs in SCI rats (Ning et al., 2019). This was visible in higher cell viability, migration, and neurotrophic factors expression *in vitro*. Additionally, the authors also noted improved locomotor function and upregulation in BDNF and NGF in the treatment group. Comparably, Seo et al. have used BM-MSCs pre-treated with low-frequency, pulsating electromagnetic fields (LF-PEMFs) to remedy a crush-injured rat mental nerve (Seo et al., 2018). The results demonstrated that injection of pre-treated BM-MSCs led to a higher count of myelinated axons, with higher overall axon density.

On top of traumatic spinal injuries, pre-treatment of MSCs with electromagnetic fields is also being tested as a novel therapeutic approach in Parkinson’s disease. A 2016. study by Jadidi et al. used LF-EMFs (50 Hz, 40 or 400 uT, 1 h/day) to treat BM-MSCs prior to their transplantation into the left ventricle of Parkinsonian rats (Jadidi et al., 2022). After 2 weeks, the injected MSCs migrated to the SNpc and differentiated into dopaminergic neurons. The rats in the treatment group exhibited significant behavioral improvements, with an increase in brain BDNF levels (Jadidi et al., 2022).

Besides pre-treatment or adjuvant application, EMFs are also being used congruently with the infusion of cells loaded with magnetic nanoparticles (MNPs). These are designed to facilitate easier cell guidance towards the injured site (Cho et al., 2013). The results of this study show that MNP-incorporated hBM-MSCs are actively being guided towards the injury site and, with that, promote significant behavioral recovery in SCI rats. Additionally, a 2020. study by Aldebs et al. has used superparamagnetic iron oxide nanoparticles (SPIONs) to assist in osteogenic differentiation of hAT-MSCs, in conjunction with direct stimulation with LF-PEMFs (Aldebs et al., 2020). The researchers designed a three-dimensional hydrogel scaffold based on self-assembled RADA16 peptides containing SPIONs, which was used to grow hAT-MSCs. Not only did they show that LP-PEMFs and SPIONs have no negative effects on cell viability, but they also demonstrated that these modalities can distinctly induce early differentiation of hAT-MSCs into an osteoblastic phenotype.

**FIGURE 4:**
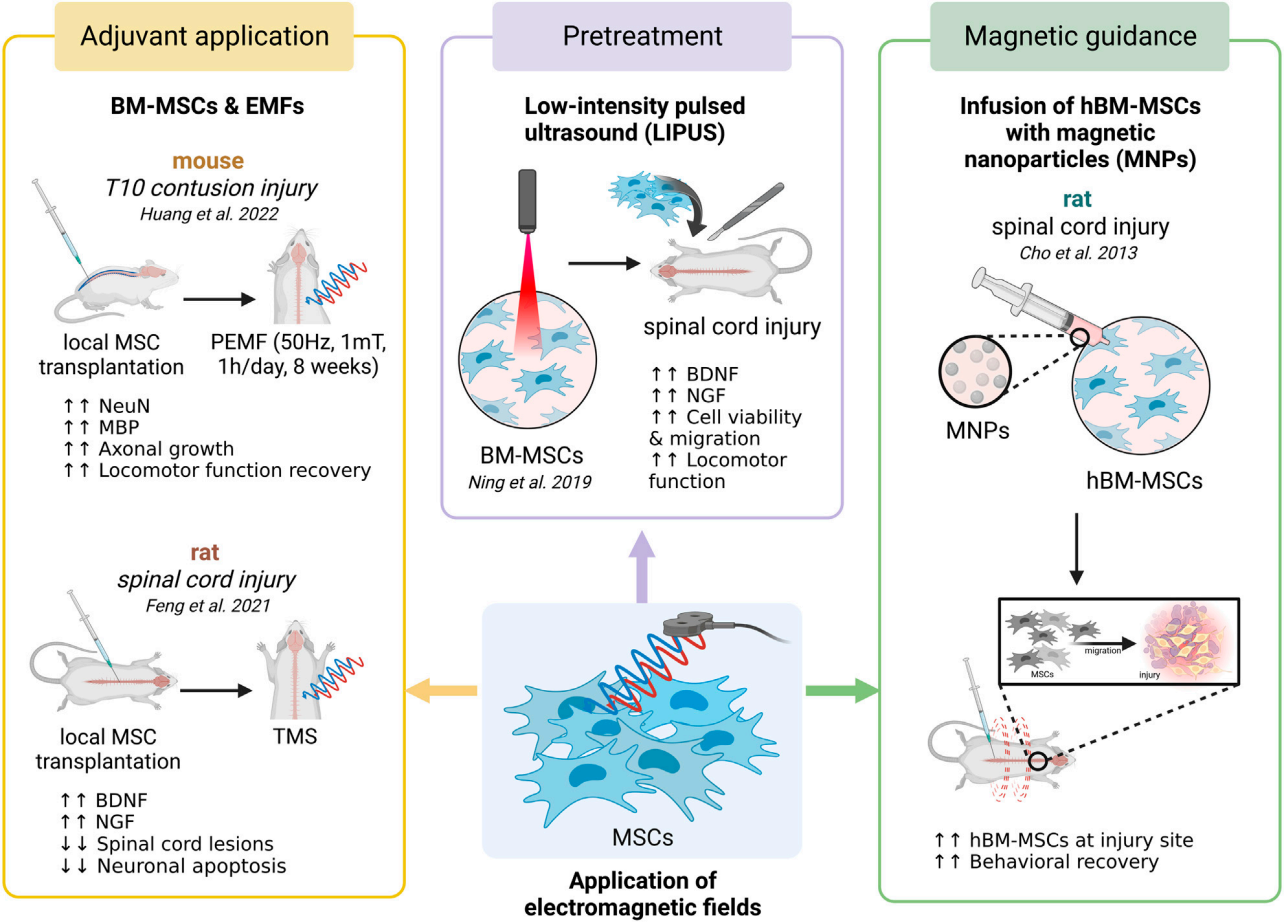
Summary of different electromagnetic field application modalities as they pertain to enhancing the beneficial effects of MSCs. *Aside from adjuvant application of MSCs and electromagnetic fields, researchers are utilizing EMF-pretreated MSCs for the purpose of enhancing their expansion rate. On the other hand, EMFs are also being used for magnetic guidance of MSCs towards the injury site following their infusion with magnetic nanoparticles (Created with* *BioRender.com* *)*.

Even though clinical trials on EMF-treated MSCs are still in their early stages and, to our knowledge, none have been performed on neural tissue, results from the aforementioned preclinical studies suggest that they appear to a safe and effective option for tissue reconstruction (Figure 4). Nevertheless, some concerns regarding their use at a therapeutic level have been raised (D’Angelo et al., 2015). These predominantly pertain to the use of specific amplitudes, frequencies, and exposure times (Miskon and Uslama, 2011).

When discussing cytotoxic and genotoxic effects of EMFs on MSCs, a multitude of studies have shown no significant changes in cells treated with fields within a specific range (Razavi et al., 2014; Fan et al., 2015; Kim et al., 2015; Ross et al., 2018; Samiei et al., 2020). For example, a 2015. study by Kim et al. reported no evidence of cytotoxicity, morphological changes or necrosis to hBM-MSCs upon exposure to EMFs (45 Hz, 1 mT, 16 h/day) (Kim et al., 2015). Interestingly, there was also no change in lactate dehydrogenase (LDH) activity in EMF-treated MSCs, suggesting no obvious damage to the cell membrane. Next, a study by Fan et al. investigated the effects of extremely low frequency electromagnetic fields (ELF-EMFs) (50 Hz, 1 mT, 4 h/day) on proliferation and cytokine production of MSCs (Fan et al., 2015). No cytotoxic effects have been reported. Similarly, Ross et al. tested the cytotoxic and genotoxic activity of ELF-EMFs (5 Hz, 0.4 mT, 20 min/day) on hBM-MSCs (Ross et al., 2018). Cytogenetic investigation into the viability and proliferation of these cells, coupled with morphological genome stability analysis, revealed no signs of cytotoxicity. Additionally, no chromosomal breakage was visible in the karyotype analysis, implying no genotoxicity. Comparable results were also obtained by Razavi et al. (Razavi et al., 2014) and Samiei et al. (Samiei et al., 2020) with no decrease in cell survival and proliferation following EMF exposure being reported. Even though none of the studies reported any cytotoxic or genotoxic effects, their findings are limited to EMF exposure within a small range of frequencies, limited length of exposure, and predetermined amplitude. As such, additional studies are needed to evaluate and quantify the effects of EMFs on MSCs as they pertain to a larger range of frequencies, longer exposure times, and a variety of different amplitudes prior. This is a vital step in facilitating translation towards clinical practice.

## 6 Discussion

Due to their wide availability through a multitude of organ systems, MSCs are among the more prominent cell types being used for tissue regeneration nowadays. Even though they boast high immunomodulatory activity, the ability to differentiate into neuron-like cells, and neuroprotective effects, they are still a heterogeneous cell population that exhibits source-dependent differences influencing their applicability. As such, an increasing number of research is focusing on genetic modification of these cells, their pre-treatment under different culture conditions, and their use as therapeutic carriers meant to facilitate targeted delivery of pharmacological agents. Even though most of the research within the field suggests that MSC-based therapies appear to be safe, some adverse effects have been reported. These include: 1) lack of immunocompatibility, 2) differentiation into undesirable cell types, 3) tumor-promoting ability, and 4) initiation of an uncontrolled immune response. To address these developing concerns, several researchers are leveraging MSCs’ secretory capabilities and investigating cell-free techniques by employing MSC-derived exosomes and EVs. Most of these have entered clinical trials, with varied amounts of success—while some lead to functional recovery in diseases such as multiple sclerosis, Alzheimer’s disease and ischemic stroke, others had no effect. Nevertheless, since ensuring efficient engraftment and survival of appropriate quantities of transplanted cells remains a challenge, a variety of novel bioengineering approaches are being developed. These mainly include the use of both natural and synthetic biomaterials as scaffolds designed to improve the retention of implanted cells. Another promising approach towards optimizing MSC-based treatment is the use of electromagnetic fields which have been shown to promote the cell’s proliferation and drive neurogenesis. These novel approaches have not yet reached clinical applications but show promise.

Even though the past decade has seen a rise in both experimental studies using animal models and clinical trials of MSC-based therapies for neurological disorders, much remains unknown. Therefore, additional research is needed to quantify and compare the risks involved in cell–based vs cell-free treatments, develop novel strategies to obtain larger quantities of healthy cells, and reduce the variability of results due to MSCs’ innate heterogeneity.
